# CD44/POU2F2/BCL9L axis mediates MIF-driven SPP1^+^TAM activation in colorectal cancer metastasis

**DOI:** 10.7150/ijbs.116575

**Published:** 2026-02-04

**Authors:** Fan Zhang, Bingquan Zhao, Xueying Fan, Zhiqiang Shi, Peilan Guo, Haibo Zhang, Hiu-Yee Kwan, Zhongqiu Liu, Tao Su

**Affiliations:** 1 State Key Laboratory of Dampness Syndrome of Chinese Medicine, The Second Affiliated Hospital of Guangzhou University of Chinese Medicine, China.; 2 State Key Laboratory of Traditional Chinese Medicine Syndrome, Guangdong Key Laboratory for Translational Cancer Research of Chinese Medicine, International Institute for Translational Chinese Medicine, Guangzhou University of Chinese Medicine, Guangzhou, Guangdong, 510006, China.; 3 Chinese Medicine Guangdong Laboratory, Guangdong Hengqin, China.; 4 Centre for Cancer & Inflammation Research, School of Chinese Medicine, Hong Kong Baptist University, Hong Kong, China.

**Keywords:** colorectal cancer metastasis, MIF, SPP1^+^TAM, POU2F2, MIF/CD44/POU2F2/BCL9L signaling axis

## Abstract

**Background:**

Tumor-associated macrophages (TAMs), especially SPP1^+^TAMs are associated with poor prognosis of colorectal cancer (CRC). However, the underlying mechanism remains unclear, and the therapeutic targets have yet to be identified.

**Methods:**

Single-cell RNA sequencing (scRNA-seq) data were used to explore the interactions between SPP1^+^TAMs and CRC cells. TAM co-culture model, liver metastasis models and clinical tissue microarray (n=42) were used to validate the key secreted cellular factor and its associated receptor that mediated the interactions between SPP1^+^TAMs and CRC cells.

**Results:**

We found that migration inhibitory factor (MIF) was the most important signaling molecule involved in the interaction between SPP1^+^TAMs and CRC cells, as revealed by cellular interaction analysis of integrated scRNA-seq datasets. Interestingly, SPP1 was co-expressed with MIF receptor CD44 on SPP1^+^TAMs. When SPP1^+^TAMs was activated, CD44 was crucial for MIF-mediated angiogenesis. Our data showed that CRC cells activated SPP1^+^TAMs, which was abolished by blocking the MIF signaling both *in vitro* and *in vivo*. Furthermore, the pathological role of MIF is suggested by the elevated expression of MIF and activation of SPP1^+^TAMs in CRC patients, as demonstrated in clinical tissue microarray. Further mechanistic studies revealed that POU2F2 was a crucial transcription factor mediating MIF-driven activation of SPP1^+^TAMs, and that BCL9L was a direct downstream target of POU2F2.

**Conclusions:**

Our findings suggest that the MIF/CD44/POU2F2/BCL9L signaling axis is involved in the proangiogenic capacity of activated SPP1^+^TAMs, thereby enhances CRC metastasis. Targeting this novel signaling axis can effectively suppress the SPP1^+^TAM activation, which represents a promising and pivotal strategy for managing CRC metastasis.

## Introduction

Colorectal cancer (CRC) is the third most prevalent malignancy worldwide and a leading cause of cancer-related mortality 1. Distant metastasis, particularly to the liver and lungs, is the primary contributor to mortality in CRC patients 2. Approximately 20% of newly diagnosed CRC patients are present with metastatic disease. Despite recent improvements in survival rates, metastatic CRC (mCRC) remains a significant clinical challenge, with a 5-year survival rate of only approximately 14% 3.

Tumor-associated macrophages (TAMs) serve as a paradigm for the understanding of the complex interplay between cancer progression and metastasis. TAMs, which predominantly originate from bone marrow-derived monocytic precursors in peripheral blood, are recruited and infiltrate into the tumor microenvironment (TME). The polarization of macrophages toward the M1 phenotype has been associated with tissue damage and destruction, as well as the elimination of tumor cells, whereas M2 polarization has been linked to cancer progression and metastasis. However, an increasing number of studies have shown that mixed phenotypes often coexist within the TME, suggesting that dichotomous M1/M2 categorization may oversimplify the transcriptionally dynamic nature of macrophages under the complexities of the TME 4. The infiltration of macrophages in tumors is generally associated with a poor prognosis 5. Nevertheless, some studies 6, 7 have suggested that increased macrophage infiltration might correlate with a better prognosis in CRC patients. The relationship between macrophages and tumor malignancy needs further study. A recent study has identified a novel macrophage marker, CXCL9:SPP1, which has superior prognostic value compared with traditional M1/M2 macrophage-associated markers 8. Meanwhile, a proangiogenic population of TAMs, specifically SPP1^+^TAMs 9, has been identified as a pivotal factor in the development of CRC 4. However, the underlying mechanism of SPP1^+^TAMs activation, and its correlation with CRC malignancy remain unclear.

Macrophage migration inhibitory factor (MIF) is a multifunctional cytokine that plays a pivotal role in the pathogenesis of various inflammatory and autoimmune diseases 10. It has also been detected in different types of cancer cells. Studies have shown that MIF contributes to the carcinogenesis by deactivating p53 and promoting angiogenesis 11. MIF hinders M1 polarization of macrophages during brain tumor development 12. MIF also promotes angiogenesis under hypoxic conditions 13, and supports tumor progression by recruiting macrophages and promoting angiogenesis in CRC 14. However, whether MIF is involved in the activation of angiogenic SPP1^+^TAMs remains unclear.

POU2F2, also known as Oct2, is a member of the POU family of transcription factors (TFs), which includes other family members such as Oct-1, Oct-2 and Oct-11 15. POU2F2 is typically overexpressed in various types of cancers, and elevated POU2F2 expression is correlated with poor prognosis in CRC patients 15. However, the molecular mechanisms underlying their pathogenic role remain largely unknown.

Given the crucial roles of SPP1^+^TAMs in CRC, we first conducted a cellular interaction analysis, in which MIF was identified as the most significant interaction signaling molecule between SPP1^+^TAMs and tumor cells. The roles of MIF in regulating SPP1^+^TAMs activation was validated in both *in vitro* and *in vivo* studies. Additionally, ATAC-seq and RNA-seq were employed to investigate the transcriptional regulatory networks involved in SPP1^+^TAMs activation. This study identifies a novel therapeutic target and provides strong scientific evidence for the development of SPP1^+^TAM-targeted therapeutic strategies to treat CRC metastasis.

## Materials and Methods

### Chemicals and reagents

Enzyme-linked immunosorbent assay (ELISA) kits including human MIF, human IL10 and human TNF-α were purchased from Linkebio Co. Ltd. (Hangzhou, China), and the mouse MIF was purchased from Meimian Bio-Engineering Co. Ltd. (Suzhou, China). Primary antibodies including anti-SPP1, anti-MARCO, anti-MIF, anti-POU2F2, anti-E-cadherin, anti-N-cadherin, anti-Vimentin, anti-MMP2, anti-GAPDH, anti-TIMP-1, anti-CD74, anti-CD44, anti-POU2F2 and anti-CXCR4 were purchased from Santa Cruz Biotechnology (Santa Cruz, CA). The anti-β-tubulin was purchased from Abmart Shanghai Co.,Ltd (Shanghai, China). Bicinchoninic acid assay (BCA) protein assay kit was purchased from Thermo Fisher Scientific (Waltham, USA). All materials for cell culture were purchased from Life Technologies Inc. (California, USA).

### Data acquisition and preprocessing

Single-cell RNA sequencing (scRNA-seq) datasets from CRC primary tumors (GSE146771) and CRC liver metastases (GSE164522) were downloaded from the GEO database. Doublets were identified and removed using Scrublet with default parameters. To mitigate batch effects, datasets were integrated using Harmony, followed by standard downstream processing using the Seurat package. Gene expression was normalized using the sctransform method. Monocyte-macrophage populations were isolated based on the expression of the canonical markers CD68 and CD163. Subpopulations were annotated using well-established gene signatures as described previously^5^: Mono-CD14 (defined by CD14, CD36, S100A8 and S100A12); Mono-CD16 (defined by CD16, FCGR3A, TCF7L2 and CX3CR1); Macro-NLRP3 (defined by NLRP3, IL1B and EREG); TAM-C1QC (identified by the markers of C1QC, C1QA, C1QB, ITM2B, APOE and CD81); and TAM-SPP1 (defined by SPP1, VCAN, TIMP1, VEGFA, MARCO and FN1). Malignant epithelial cells were identified using inferCNV to detect somatic copy-number alterations (SCNAs). Cell distributions were visualized using uniform manifold approximation and projection (UMAP) analysis. The proportion of each cell subpopulation was calculated as the number of cells in that subpopulation divided by the total number of retained cells.

### Identification of significant ligand‒receptor pairs

To identify significant ligand‒receptor cell interaction signals between tumor cells and macrophages, we applied CellChat (version 1.5) 16 using its built-in ligand‒receptor database CellChatDB. Following the standard CellChat workflow, significant ligand‒receptor pairs were then identified through perturbation testing, and their related pathways were inferred. We screened interactions between tumor cells and macrophages, and further computed and visualized the network centrality scores to identify potential signaling pathways related to SPP1^+^TAM activation.

### Gene set enrichment analysis

Gene set variation analysis was performed with the GSVA package (version 1.3.0). The gene sets we used for SPP1^+^TAMs functional analysis were exported using the GSEABase package (version 1.44.0). The differences in pathway GSVA scores per cell among Mono-CD14, Mono-CD16, TAM-SPP1, Macro-NLRP3 and TAM-C1QC cluster were calculated with LIMMA package (version 3.37.11) as previously described 4. The gene set used for SPP1^+^TAMs activity GSVA score calculation was obtained from previous study 4. M1 and M2 signature scores were calculated using add AddModuleScore() function from Seurat as previously described 4.

### Survival analysis

Transcriptomic and clinical data for the TCGA COAD (colon adenocarcinoma) and READ (rectal adenocarcinoma) cohorts were obtained from the UCSC Xena platform. Additionally, publicly available CRC cohorts from the Kaplan‒Meier (KM) plotter database (https://kmplot.com/analysis) were integrated into the analysis. The optimal cutoff for survival stratification was determined using the survminer R package. Survival analyses were conducted using univariate Cox proportional-hazards regression models implemented in the R survival package. KM survival curves were generated and visualized using the ggsurvplot package.

### RNA library preparation, sequencing and data preprocessing

Total RNA from the cell samples was extracted according to the instruction manual of the TRIzol reagent (Life technologies, California, USA). The RNA concentration, purity and integrity were measured via a NanoDrop 2000 (Thermo Fisher Scientific, Wilmington, DE). A total amount of 1 μg RNA per sample was used as input material for the RNA sample preparations. The sequencing libraries were generated using a Hieff NGS Ultima Dual-mode mRNA Library Prep Kit for Illumina [Yeasen Biotechnology (Shanghai) Co., Ltd.] following the manufacturer's recommendations. The libraries were subsequently sequenced on an Illumina NovaSeq platform to generate 150 bp paired-end reads (PE150). Raw fastq data were first aligned to the hg38 genomes using STAR after the adapter was removed. DEGs were analyzed via DEseq2. DEGs with *Benjamini-Hochberg* multiple comparison adjustment with an FDR threshold of < 0.001 and log2-fold-change threshold of at least three were considered significant.

### ATAC-seq data analysis

The ATAC-seq data were processed according to a previously described protocol 17. The raw fastq data were subjected to prealignment quality control via FastQC and cutadapt 18. After read trimming, those data were mapped to GRCh38 using Bowtie2 genome to generate bam files. Unmapped unpaired reads, the mitochondrial genome 19 in ENCODE blacklisted regions (https://sites.google.com/site/anshulkundaje/projects/blacklists) and PCR duplicated reads 20 were removed.

To identify the differentially accessible chromatin regions (DARs) between two samples according to the ATAC-seq data, we used a slicing window-based method in Csaw to perform differential tests 21. To identify the sequence motif enriched in the ATAC-seq peaks, findMotifsGenome.pl from the HOMER program (default parameters) was used. Peak annotation was performed using ChIPseeker 22.

De novo transcription factor binding footprints were identified using HINT-ATAC 23. To construct the transcription factor regulatory network associated with TAM activation, we integrated the RNA-seq data to identify TAM-activated transcription factors and intersected with the transcription factor binding footprints identified in the TAM ATAC-seq data. On the basis of the positions of the transcription factor binding footprints and their nearest gene transcription start site (TSS), we constructed a transcription factor regulatory network and visualized it via Cytoscape.

### ATAC-seq library preparation

ATAC-seq was performed by Biomarker Technologies Biotechnology Ltd. according to ATAC-seq protocol 24. ATAC-seq libraries for THP-1 cells (RRID:CVCL_0006) were prepared following a previously described protocol 25. Briefly, samples were lysed in lysis buffer containing Tris-HCl, NaCl, MgCl_2_, and NP-40. Nuclei were isolated by centrifugation after lysis and then incubated with Tn5 transposase and tagmentation buffer at 37°C for 30 minutes. The tagmentation reaction was stopped by the addition of stop buffer. PCR analysis was performed to amplify the library using 1X NEBNext High-Fidelity PCR Master Mix (New England Biolabs, MA), followed by purification using AMPure beads. These samples were finally subjected to sequencing on an Illumina NovaSeq 6000 platform via a 150 bp paired-end sequence approach.

### Spatial cell type mapping via spatial transcriptomics

We utilized the Cell2location package 26 to map the SPP1^+^TAMs single-cell cluster that was identified in both the primary site and liver metastasis site of CRC tissues from the GSE164522 dataset to a spatial transcriptomics dataset from a previous study on CRC liver metastases 27. Briefly, we first employed negative binomial (NB) regression to estimate the reference cell type signatures of SPP1^+^TAMs in GSE164522, followed by model training and quality control including reconstruction accuracy and model loss assessment. Finally, we utilized the trained model to map SPP1^+^TAMs in both the primary CRC site and liver metastasis site of CRC using a spatial transcriptomics dataset.

### Chromatin immunoprecipitation (ChIP) and quantitative PCR analysis

Cells were fixed with 1% methanol-free formaldehyde and lysed in SDS buffer. ChIP assays (P2078, Beyotime) were performed according to the manufacturer's instructions, using 1 μg of specific antibodies or IgG as a negative control. The complexes were subsequently washed in sequential buffers and eluted with SDS/NaHCO_3_, followed by reverse cross-linking at 65°C for 4 h. DNA was extracted with phenol-chloroform and analyzed by qPCR using specific primers. The qPCR program was as follows: 95°C for 10 min, followed by 45 cycles of 95°C for 15 sec and 60°C for 1 min.

### Cell culture

The human monocyte cell line THP-1, human normal colon epithelial cell line NCM460, and CRC cell lines (HCT116 and LOVO) were obtained from the Chinese Academy of Sciences in Shanghai (Shanghai, China). Cells were cultured in RPMI 1640 medium (Gibco, USA) supplemented with 10% fetal bovine serum (FBS) (Gibco, USA) at 37 °C in a humidified atmosphere with 5% CO_2_. To generate macrophages, 3 × 10^5^ of THP-1 cells were seeded and treated with 200 nM phorbol 12-myristate 13-acetate (PMA) (Sigma‒Aldrich, USA) for 24 h to induce differentiation into macrophages. The expression of the macrophage marker CD68 was determined using RT‒qPCR to confirm successful differentiation.

### CRC cell‒TAM coculture system

CRC cells were seeded in a 6-well plate and cultured in 2 mL of serum-free RPMI 1640 for 24 h. The conditioned medium was then collected and centrifuged at 1,000 rpm for 5 min. PMA-induced THP-1 macrophages (3 × 10^5^ cells) were plated in a 6-well plate. Next, 1 mL of CRC-conditioned medium was mixed with 1 mL of macrophage culture medium. To generate TAMs, the culture medium of PMA-induced THP-1 macrophages was replaced with 2 mL of the mixed medium, and the cells were incubated for an additional 48 h. The morphologies of the treated macrophages were observed and photographed under an inverted microscope. To explore the interaction between TAMs and CRC cells, the cocultivation of TAMs and CRC cell lines was conducted using a noncontact coculture transwell system, which contained two-chamber dishes to allow the exchange of soluble diffusible factors while preventing their direct contact. After 24 h of coculture, CRC cells were harvested for further analysis.

### *In vitro* tube formation assay

To evaluate the pro-angiogenic potential of TAMs, a tube formation assay was conducted using human umbilical vein endothelial cells (HUVECs). Briefly, serum-free conditioned medium was collected from induced TAM cultures after a 24-hour incubation. Subsequently, HUVECs were seeded onto Matrigel-coated plates and cultured for 4-6 hours in the corresponding TAM-conditioned medium. Tube formation was visualized and imaged using an inverted phase-contrast microscope.

### Western blotting

Western blot analysis was performed by lysing cells with RIPA buffer supplemented with a protease inhibitor cocktail (Thermo Scientific, USA). The protein samples were resolved on SDS‒PAGE gels and subsequently transferred onto PVDF membranes (Millipore, USA). Following blocking with 5% BSA, the membranes were incubated overnight at 4 °C with primary antibodies. HRP-conjugated secondary antibodies were then applied and incubated for an additional 2 h of incubation at room temperature. Protein bands were visualized using the Bio-Rad ChemiDoc XRS+System, and densitometric analysis was performed using Bio-Rad Image Lab software.

### ELISA

The levels of TNF-α, IL10 and MIF in mouse serum or culture medium were measured by ELISA kits according to the manufacturer's instructions.

### Generation of a stable MIF knockdown cell line via lentivirus transfection (Lv) transfection

The GV493 vector lentiviral RNAi expression system Lv-shRNA-MIF was used to construct lentiviral shRNAs for gene knockdown, which were designed, constructed and synthesized by Shanghai GeneChem Co., Ltd. The sequence of Lv-MIF-shRNA was CCGGCCAGAACCGCAACTACAGTAACTCGAGTTACTGTAGTTGCGGTTCTGGTTTTT and targeted the *Mus musculus* MIF gene sequence CCAGAACCGCAACTACAGTAA. To establish a stable CT26 Lv-shRNA-MIF cell line, CT26 cells were initially seeded at a density of 3×10^3^ cells per well in a 24-well plate and allowed to adhere for 24 h before infection with lentivirus. The intensity of green fluorescent protein (GFP) fluorescence was used to quantify the transduction efficiency. The stably transfected clones were screened with puromycin at concentrations ranging from 5-10 μg/mL.

### Animal experiments

Male 6-week-old BALB/c mice were purchased from the Laboratory Animal Center of Guangdong [SCXK(GZ)2022-0002, Guangzhou, China]. They were kept in the animal laboratory at International Institute for Translational Chinese Medicine [SYXK(GZ)2024-0144]. **CRC liver metastasis model:** BALB/c mice were anesthetized with isoflurane and intrasplenically injected with 4 × 10^6^ CT26-luciferase cells in 100 μL PBS to establish a CRC liver metastasis model (n=6). The extent of metastasis was assessed by monitoring tumor burden in the liver.

**CRC lung metastasis model:** approximately 5 × 10^6^ CT26-luciferase cells in 100 μL PBS were injected into the tail vein of male 6-week-old BALB/c mice (n=6). Mice were closely monitored for respiratory symptoms to evaluate metastatic progression.

After being sacrificed, metastases in the livers or lungs were quantified using an IVIS Lumina XRMS Series III (PerkinElmer, MA, USA), and image analysis was performed with Living Image software 4.4. Fluorescence data were presented as photon flux (p/s). All the animal experiments were approved by the Ethics Committee in International Institute for Translational Chinese Medicine (Ethical Review No. 20230812).

### RNA interference

siRNAs, including *Homo sapiens* POU2F2 siRNA, CD44 siRNA, CD74 siRNA and CXCR4 siRNA, were designed and chemically synthesized by GenePharma (Suzhou, China). THP-1 cells were seeded in 6-well plates and treated with 200 nM PMA for 24 h to induce their differentiation into macrophages. Subsequently, cells were transfected with 6.25 pmol/ml siRNA using 1 μl/mL Lipofectamine for 6 h. After transfection, the culture medium of PMA-induced THP-1 macrophages was replaced with 2 mL of CRC-conditioned medium to established a TAM co-culture model, and followed by an additional 72 h of induction culture.

### Immunofluorescence staining

Tissue samples were fixed in 10% buffered formalin for 4 to 24 h, dehydrated through a graded ethanol series, and embedded in paraffin. Sections (5 μm) were cut using a rotary microtome, followed by deparaffinization and rehydration. Non-specific binding was blocked with PBS containing 0.3% Triton X-100 and 10% goat serum. Sections were incubated overnight with primary antibodies, followed by fluorescently labeled secondary antibodies and nuclear staining with DAPI. Fluorescence images were acquired using a microscope.

### Dual luciferase reporter assay

The human transcription factor POU2F2 overexpression vector was constructed by inserting the coding sequence (CDS: chr11: 118913018-118913355) into the eukaryotic expression vector pcDNA3.1, generating the POU2F2 overexpression plasmid provided by Suzhou Jima Gene Co., Ltd. For the dual luciferase reporter assay, 293T cells and THP-1cells were transfected following the protocols provided by Suzhou Jima Gene Co. Briefly, 1 μg of the GPL4-BCL9L-promoter plasmid containing the BCL9L promoter or the GPL4-NC control plasmid was added to another 50 μl of serum-free medium and mixed. Additionally, 1 μg of the POU2F2 overexpression plasmid pcDNA3.1-POU2F2-OV or the corresponding control plasmid pcDNA3.1-NC was included. After allowing the mixture to sit at room temperature for 5 minutes, the two components were combined and incubated at room temperature for an additional 20 min. Finally, the complex was added to the cell culture medium at a final concentration of 2 ng/μL. Relative luciferase activity was measured using a Dual Luciferase Reporter Assay Kit (Biyuntian Biological Co., Ltd.) according to the manufacturer's instructions. Finally, relative light unit (RLU) values were calculated.

### Clinical human tissue microarray

Human tissue microarray slides were procured from Shanghai Outdo Biotechnology Company Ltd. The arrays included 60 tissue samples including those from colon tumors, metastatic tissue and adjacent normal tissue microarrays (HColA060CD01). The tissue samples were provided by the National Human Genetic Resources Sharing Service Platform (2005DKA21300). All participants provided written informed consent, ensuring compliance with the platform's guidelines, and their identity and privacy were completely safeguarded. Additionally, the study protocol received approval from the Ethics Committee of Chengdu Medical College and adheres to the principles outlined in the Declaration of Helsinki.

### Statistical analysis

Data were organized using Microsoft Excel 2019 and statistically analyzed with SPSS version 17.0 software. Experimental data that were normally distributed are presented as mean ± standard deviation (SD), whereas data that were not normally distributed are presented with the interquartile range. For independent samples, if the data passed the *Shapiro* test for normal distribution or the *Bartlett* test for homogeneity of variance, an independent samples t-test was employed for comparisons between two groups, and one-way analysis of variance (ANOVA) followed by Tukey's honest significant difference (HSD) test was used for multiple comparisons. The *Bonferroni* correction was applied for *P-*value calculations. When data did not meet the criteria for normal distribution or homogeneity of variances, the *Kruskal‒Wallis* test was used for comparisons among multiple groups, with the *Nemenyi* test for post-hoc multiple comparisons and the *Bonferroni* correction for *P*-value calculations.

## Results

### MIF is the key signaling molecule mediating interaction between CRC cells and SPP1^+^TAMs

**Figure 1:**
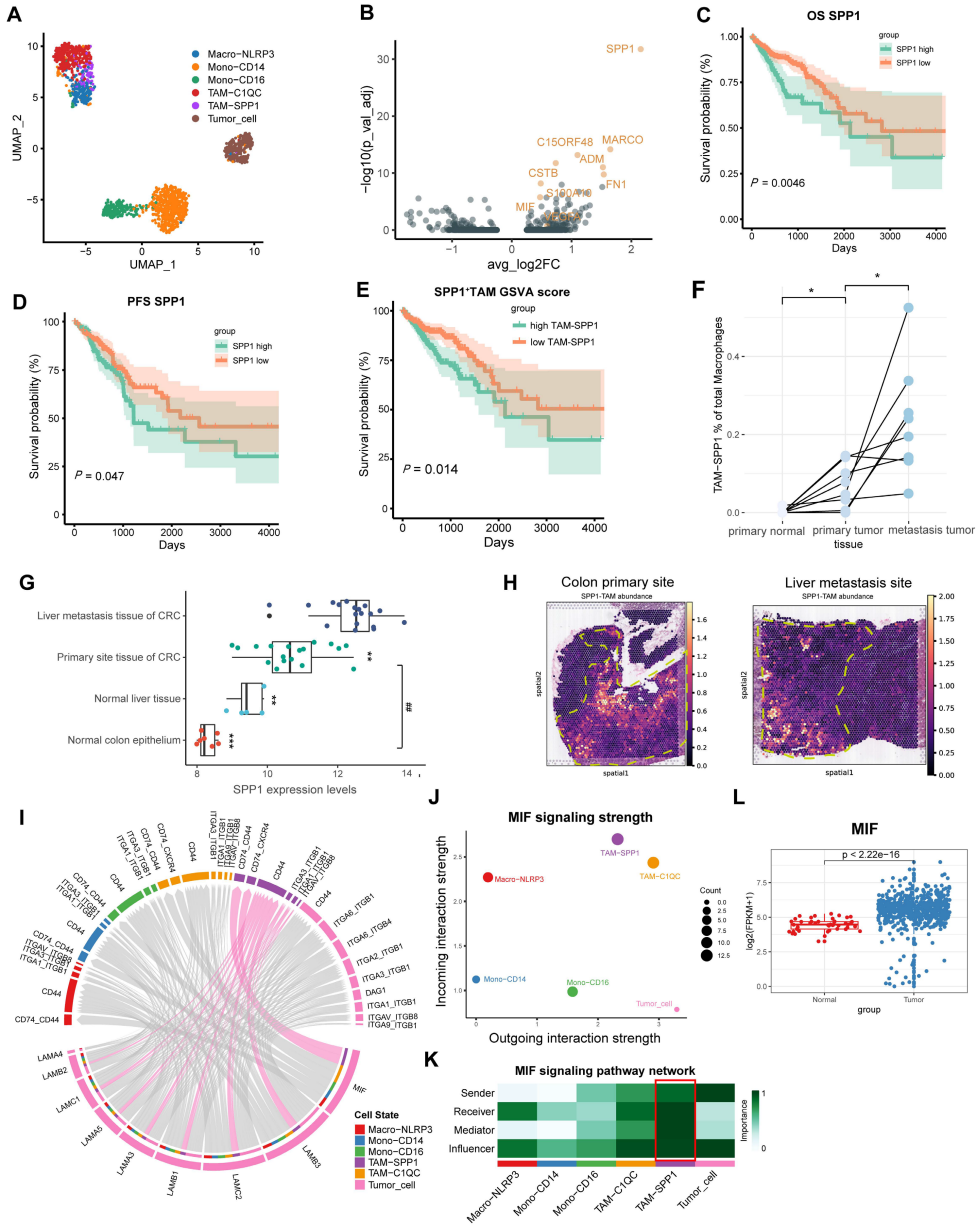
** MIF is the most significantly interacting signaling between CRC cells and SPP1^+^TAMs. (A)** UMAP plot illustrating the clustering of monocytes, macrophages and tumor cells from the GSE146771 dataset. **(B)** Volcano plot illustrating the differential expression analysis of specific marker genes of SPP1^+^TAMs. **(C)** Kaplan-Meier analysis of patients' overall survival based on SPP1 expression in the READ and COAD cohorts from the TCGA database. **(D)** The Kaplan-Meier analysis of the patients' progression-free survival (PFS) based on SPP1 expression within the READ and COAD cohort from the TCGA database. **(E)** Kaplan-Meier analysis of patients' overall survival curves according to the SPP1^+^TAMs signature GSVA score within the READ and COAD cohort from TCGA database. **(F)** The dot plot illustrating the proportion of SPP1^+^TAMs within macrophages across paired tissue samples, including primary normal colon tissues, primary tumor tissues, and liver metastatic sites. *P<0.05, *vs.* primary normal tissues, ^#^*P*<0.05, *vs.* metastasis tumor tissues (n=8). **(G)** Boxplot illustrating the SPP1 expression in normal colon (n=7) and liver tissues (n=5), as well as primary CRC (n=18) and liver metastatic tissue (n=18) within the GSE14297 dataset. *P<0.05, **P<0.01, ***P<0.001 *vs.* liver metastasis tissue of CRC; ^##^*P*<0.01, *vs.* normal colon epithelium. **(H)** Spatial mapping of SPP1^+^TAMs cellular architecture at the primary site and liver metastasis site using integrated single cell and spatial transcriptomics data. **(I)** Chord diagram showing the outgoing signaling pathways of the tumor cells and its corresponding receptors and ligands. The ligands released by tumor cells are situated at the lower part of the diagram, while the receptors at the upper part are indicated by the colors specified in the legend. Lines demonstrate the interaction between the signal and SPP1^+^TAMs. **(J)** Scatter plot illustrating the strength of incoming and outgoing signaling in the MIF pathway within tumor cells and mononuclear cells. **(K)** Analysis of cell‒cell interaction network centrality scores to visualize the role of signaling among tumor cells and mononuclear cells. **(L)** Boxplot depicting the expression of MIF in the READ and COAD cohorts from the TCGA database.

Two CRC scRNA-seq datasets, GSE146771 (CRC clinical samples) and GSE164522 (CRC liver metastasis clinical samples) were used to explore the mechanism and targets of CRC metastasis. First, we followed the Seurat workflow using the sctransform-based normalization method 28, 29. Based on the cellular markers previously reported 5, we identified and annotated the mononuclear macrophage clusters (**Figure 1A**, **Figure S1A-B**). SPP1^+^TAMs markers, such as SPP1 and MARCO were identified using Seurat's FindMarkers function (**Figure 1B**).

SPP1 was most highly expressed in the TAM and cDC clusters, with the highest levels in SPP1^+^TAMs (**Figure S1A**). Next, we performed Kaplan-Meier survival analysis using the COAD and READ datasets from The Cancer Genome Atlas (TCGA). We found significant correlations between increased infiltration of SPP1^+^TAMs and poorer prognosis in CRC patients (**Figure 1C-1E**). Consistently, integration of the GSE41568 microarray dataset revealed significantly higher SPP1 expression and SPP1^+^TAMs signature score in metastatic CRC tissues compared with nonmetastatic CRC tissues (**Figure S1C-S1D**). Furthermore, in the scRNA-seq dataset GSE164522, the proportion of SPP1^+^TAMs was significantly higher in CRC liver metastatic sites than at primary tumor sites and in normal colon tissue from matched patient (**Figure 1F, S1E-S1F**).

Analysis of the CRC liver metastasis dataset GSE14297 also confirmed the significant upregulation of SPP1 at the site of CRC liver metastasis (**Figure 1G**). Additionally, gene set variation analysis (GSVA) enrichment analysis revealed that SPP1^+^TAMs was associated with the positive regulation of endothelial cell chemotaxis, as well as the activation of the corresponding VEGF signaling (**Figure S1G**). The M1 and M2 signatures were compared across multiple monocyte and macrophage clusters. SPP1^+^TAMs exhibited a mixed M1/M2 phenotype with high M1 and M2 signature scores (**Figure S1H-S1I**). To elucidate the morphological localization and spatial distribution of SPP1^+^TAMs, we integrated the scRNA-seq data of the CRC liver metastasis samples from GSE14297 and spatial transcriptomics data from previous studies 27. We found that SPP1^+^TAMs were predominantly localized in the muscularis layer of the intestinal tumor site (**Figure 1H**). In the context of CRC liver metastasis, SPP1^+^TAMs are also distributed at metastatic tumor sites (**Figure 1H**). These findings suggest that SPP1^+^TAMs play crucial roles in CRC metastasis.

To further elucidate the interplay between SPP1^+^TAMs and tumor cells, we utilized CellChat 16 to analyze intercellular communication involving monocytes, macrophages, and tumor cells. The results indicated that the MIF signaling pathway, which belongs to the secreted signaling, exhibited the strongest communication potential in the interaction between tumor cells and SPP1^+^TAMs (**Table S1**). MIF is secreted by tumor cells (**Figure 1I**), and its signals are predominantly received by SPP1^+^TAMs, which exhibit the greatest incoming strength (**Figure 1J**). To further investigate the intercellular communication network of the MIF signaling pathway between these cell clusters, we calculated and visualized network centrality scores, and identified the critical nodes within the network. Results demonstrated that SPP1^+^TAMs played important roles as mediators and influencers in the MIF signaling network; whereas tumor cells serve as important senders and influencers (**Figure 1K**). More importantly, high expression levels of MIF were detected in COAD and READ samples from the TCGA dataset (**Figure 1L**).

### MIF levels are increased in the serum of metastatic CRC (mCRC) mouse models

**Figure 2:**
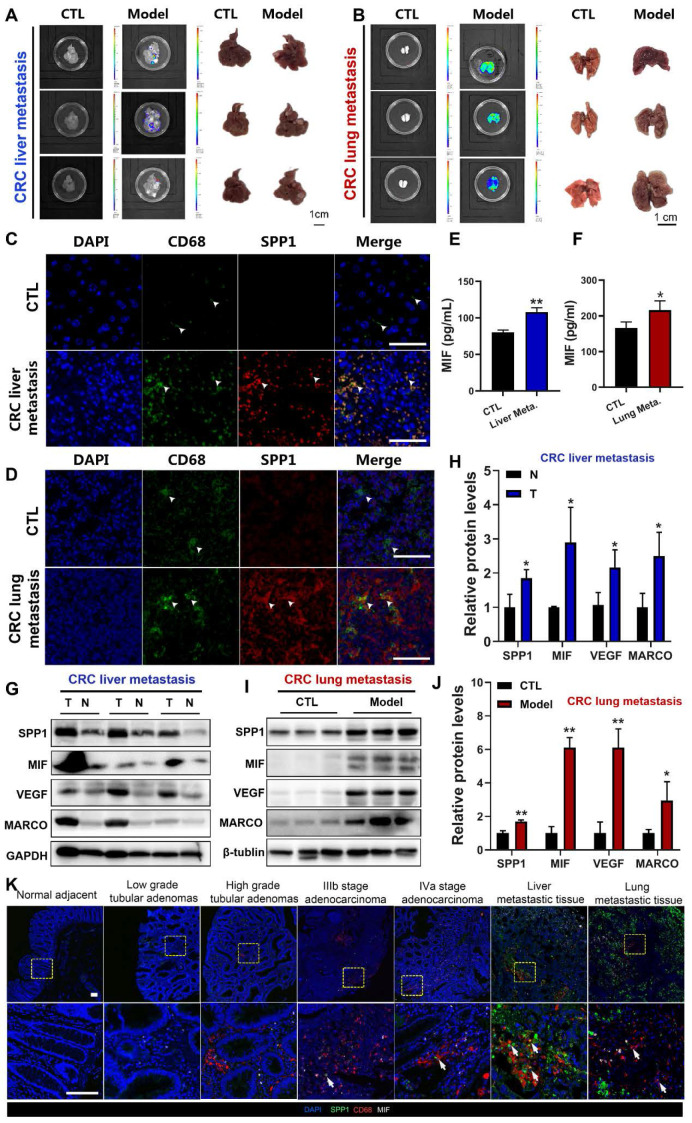
** Activation of SPP1^+^TAMs was observed in CRC liver/lung metastasis models, as well as clinical CRC tissue samples. (A)** Representative images and luminance signals showing the progression of CRC liver metastasis and control mice (n=5). **(B)** Representative images and luminance signals showing the established CRC lung metastasis model (n=5).** (C)** Immunofluorescence images of the tumors in liver tissues from the CRC liver metastasis mice and the normal liver tissues from control mice (scale bar =50 µm). **(D)** Immunofluorescence images of the tumors in lung tissues from the CRC lung metastasis mice and the normal lung tissues from control mice (scale bar =50 µm). **(E-F)** The levels of MIF in the serum of mice with CRC liver metastasis **(E)** and lung metastasis **(F)** were determined using the ELISA assay, respectively. *P<0.05, **P<0.01, *vs.* CTL group (n=4).** (G-H)** The protein levels of SPP1^+^TAMs-related markers including SPP1, MIF, VEGF and MARCO in tumor and adjacent normal tissues of the CRC liver metastasis mice were determined by using Western blotting** (G)**; and quantitative results were analyzed using Image J software** (H)**. *P<0.05, **P<0.01, *vs.* Corresponding CTL (n=4). **(I-J)** Protein levels of SPP1^+^TAMs-related markers including SPP1, MIF, VEGF and MARCO in control and lung tissues of the CRC lung metastasis mice were determined by using Western blotting** (I)**; and quantitative results were analyzed using Image J software** (J)**. *P<0.05, **P<0.01, *vs.* CTL (n=3). **(K)** Representative immunofluorescence imaging showing the expression of MIF and abundance of SPP1^+^TAMs in clinical samples from different stages of CRC (scale bar =100 µm). N: adjacent normal tissue; T: tumor tissue.

To further explore the correlation between SPP1^+^TAMs activation and CRC metastasis* in vivo*, we established CRC liver metastasis mouse models by injecting CT26 cells into the spleen (**Figure 2A**), and CRC lung metastasis mouse models by injecting CT26 cells into the tail vein (**Figure 2B**). Immunofluorescence analysis at the liver metastasis site revealed an extensive infiltration of CD68^+^macrophages (**Figure 2C**). Furthermore, immunofluorescence colocalization analyses of CD68 and SPP1 demonstrated abundant SPP1^+^TAMs in CRC liver metastatic tumors but not in normal liver tissues (**Figure 2C**). Similarly, SPP1^+^TAMs were also observed in lung metastatic sites but absent in lungs from control group (**Figure 2D**). Moreover, we also observed a significant elevation of MIF in the serum of both CRC metastasis mouse models compared with those in the control groups (**Figure 2E-2F**). The representative SPP1^+^TAM markers such as SPP1, MARCO, VEGFA and MIF 4 were significant upregulated at the protein levels in liver and lung metastatic cancer tissues compared with those in adjacent normal liver tissues (**Figure 2G-2H**) or lung samples from control group (**Figure 2I-2J**). Moreover, in clinical tissue microarray (**Table S2**), immunofluorescence showed stage-dependent increase in MIF expression and SPP1^+^TAMs infiltration, with prominent SPP1^+^TAMs accumulation in both liver and lung metastasis (**Figure 2K**). These results suggest a correlation between MIF expression and the abundance of SPP1^+^TAMs during CRC progression.

### MIF is essential for the activation of SPP1^+^TAMs in CRC metastasis mouse models

**Figure 3:**
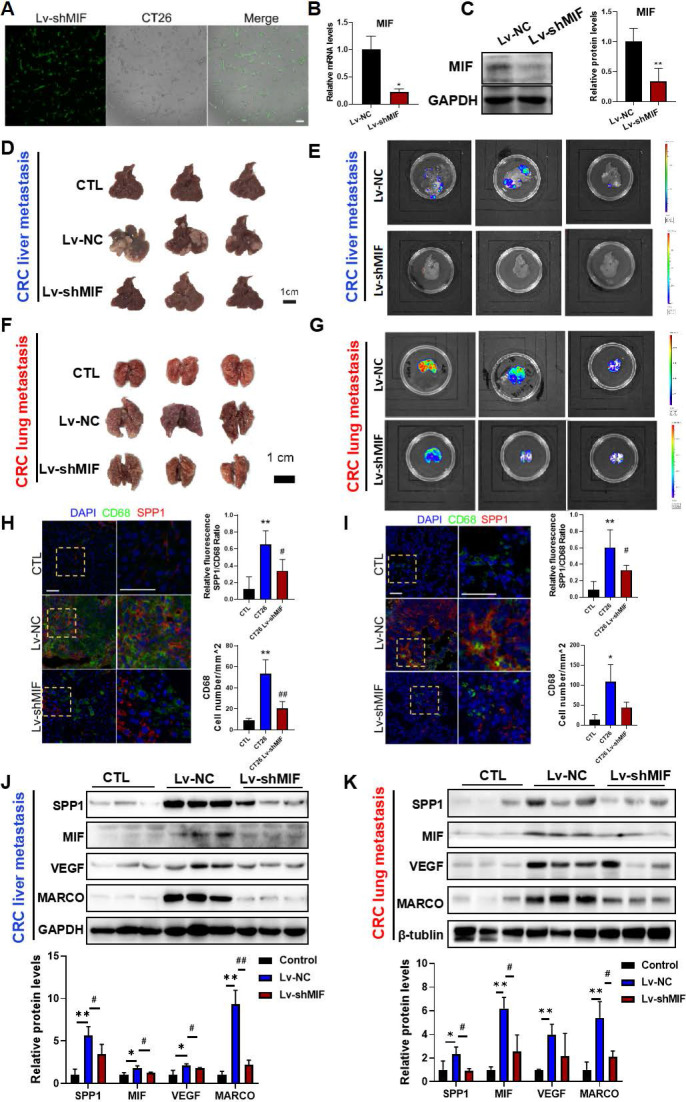
** MIF is essential for the activation of SPP1^+^TAMs in CRC metastasis models. (A-C)** The knockdown efficiency of Lv-shMIF transfected CT26 cells was confirmed by the assessment of GFP fluorescence, RT-qPCR and Western blot analyses. Representative fluorescence images of Lv-shMIF transfected CT26 cells **(A)**. The mRNA and protein levels of MIF before and after transfection were determined by using the RT-qPCR **(B)** and Western blotting **(C)**. Data are shown as Mean ± SD. *P<0.05, *vs.* Lv-NC (n=3). **(D-E)** Representative images **(D)** and fluorescence signal **(E)** of liver tissues from the CRC liver metastasis mice in Lv-NC and Lv-shMIF groups (n=5).** (F-G)** Representative images **(F)** and fluorescence signal **(G)** of lung tissues from the CRC lung metastasis mice in Lv-NC and Lv-shMIF groups. **(H-I)** Representative immunofluorescence images and statistical results of fluorescence intensity of liver **(H)** and lung **(I)** metastasis model mice showing the abundance of SPP1^+^TAMs in each group (n=4) (scale bar =50 µm). **(J-K)** The protein levels of SPP1^+^TAMs-related markers including SPP1, MIF, VEGF and MARCO in the liver **(J)** and lung **(K)** tissues of control, Lv-NC and Lv-shMIF groups were determined by using Western blotting (upper panel); and quantitative results were analyzed using Image J software (lower panel). Data are shown as Mean ± SD. *P<0.05, **P<0.01, *vs.* Control; ^#^*P*<0.05, ^##^*P*<0.01, *vs.* Lv-NC CT26 (n=3). GFP, Green fluorescent protein.

To investigate whether MIF signaling is involved in SPP1^+^TAMs activation, we established CT26 cells with stable MIF knockdown using shRNA-MIF (Lv-shMIF cells). Knockdown efficiency was confirmed by GFP fluorescence, Western blotting and RT-qPCR analysis (**Figure 3A-3C**). We then used Lv-shMIF CT26 cells to separately establish CRC liver metastasis and CRC lung metastasis mouse models. Compared with Lv-NC control group, the metastatic capabilities of tumors in the Lv-shMIF CT26 group were significantly reduced, as demonstrated by the lower fluorescence intensities in the liver (**Figure 3D-E**) and lung (**Figure 3F-G**). These results suggest that MIF plays an important role in CRC metastasis. Next, we further investigated whether MIF knockdown could reduce SPP1^+^TAMs activation in the metastasis mouse models. We found that the number of infiltrating CD68^+^macrophages was lower in the Lv-shMIF group than in the Lv-NC group (**Figure 3H-I**). Additionally, a notable decrease in the number of SPP1^+^TAMs was also observed in the Lv-shMIF group compared to the Lv-NC group in both CRC liver metastasis and CRC lung metastasis mouse models (**Figure 3H-I**). Meanwhile, we also found that the expression of SPP1^+^TAMs markers including SPP1, VEGF and MARCO were significantly lower in the Lv-shMIF CT26 group than in Lv-NC group (**Figure 3J-K**). Furthermore, triple immunofluorescence staining was performed on liver tissues from the indicated experimental groups, using CD31 to label blood vessels and co-staining CD68 and SPP1 to identify SPP1^+^TAMs. Compared with controls, the CRC liver metastasis model showed significant increase in both SPP1^+^TAM infiltration and CD31^+^ blood vessels density. Notably, in the MIF-knockdown model, both SPP1^+^TAMs number and CD31^+^ blood vessels density were significantly reduced (**Figure S2A**). Consistent with these *in vivo* findings, a HUVEC tube formation assay demonstrated that TAMs markedly promoted angiogenesis, whereas inhibition of MIF signaling with the inhibitor 4-IPP significantly suppressed this effect (**Figure S2B**). Collectively, these results not only demonstrated the pro-angiogenic capacity of SPP1^+^TAMs, but also confirmed the pivotal role of MIF in this process, suggesting that MIF drives SPP1^+^TAMs-mediated angiogenesis and promotes CRC metastasis.

### MIF enhances the migratory capacity of CRC cells and induces SPP1^+^TAMs activation *in vitro*

**Figure 4:**
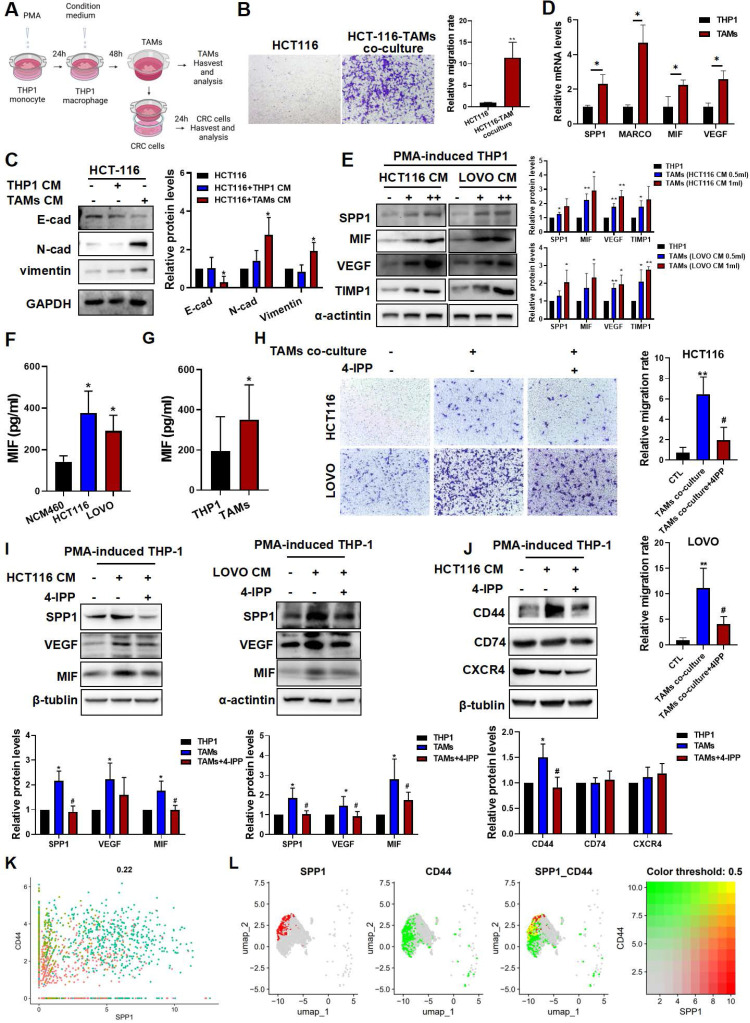
** The MIF inhibitor 4-IPP blocked MIF-mediated SPP1^+^TAMs activation *in vitro*. (A)** Schematic diagram demonstrates the experimental set up for CRC cells-TAMs co-culture system. Human monocyte THP-1 was first PMA treat for 24 h, and then induced into TAMs by treated with the CRC cells conditioned medium for another 48h. CRC cells were co-cultured with TAMs using co-culture system for 24 h.** (B)** Representative photographs of HCT-116 cells migration in the co-culture system (left panel); and quantitative results were analyzed using Image J software (right panel). Pictures were taken at 24 h after co-culture. **P<0.01 *vs.* the corresponding control (n=3).** (C)** The protein levels of EMT markers in HCT-116 cells after co-cultured in conventional medium, THP-1-CM and TAMs-CM, were determined by Western blotting, respectively. *P<0.05, *vs.* HCT116+THP-1 CM (n=3). **(D)** The mRNA levels of SPP1^+^TAMs markers in THP-1 macrophage and TAMs were determined by RT-qPCR analysis. *P<0.05, **P<0.01, *vs.* THP-1 group (n=3).** (E)** The protein levels of SPP1^+^TAMs markers in THP-1 macrophages after cultured in conventional medium or CRC-CM were determined by Western blotting, respectively. *P<0.05, **P<0.01 *vs.* control (n=3). **(F)** The levels of MIF in the conditioned medium from NCM460, HCT116 and LOVO cells were determined using the ELISA assay. *P<0.05, *vs.* NCM460 (n=3). **(G)** The contents of MIF in the cultured medium of THP-1 macrophages and TAMs were determined using the ELISA assay. *P<0.05, *vs.* THP-1 (n=4).** (H)** Representative photographs of CRC cells migration in the co-culture system after treated with 4-IPP (left panel); and quantitative results were analyzed using Image J software (right panel). Pictures were taken at 24 h after CRC cells-TAMs co-culture. Data are shown as mean ± SD. **P<0.01 *vs.* control. ^##^*P*<0.01 *vs.* CRC cells-TAMs co-culture (n=3).** (I)** The protein levels of SPP1^+^TAMs markers in THP-1 macrophages after cultured in conventional medium, CRC-CM or CRC-CM + 4-IPP were determined by the Western blotting, respectively.** (J)** The protein levels of MIF receptor in THP-1 macrophages after cultured in conditioned medium, CRC-CM or CRC-CM + 4-IPP were determined by the Western blotting, respectively. Data are shown as Mean ± SD. For (I) and (J), *P<0.05, **P<0.01, *vs.* THP-1; ^#^*P*<0.05, ^##^*P*<0.01, *vs.*TAMs. **(K)** Scatter plot showing the co-expression of SPP1 and CD44 in macrophages population. **(L)** UMAP plot showing the co-localization of SPP1 and CD44 expression in macrophages population from the scRNA-seq data.

To further investigate the molecular mechanism underlying of SPP1^+^TAM activation, we established an* in vitro* CRC cell-TAMs coculture model. THP-1 human monocytes were treated with PMA for 24 h to induce macrophage differentiation, which was confirmed by a significant increase in CD68 expression (**Figure S3A**). Subsequently, THP-1 macrophages were cultured in CRC cell conditioned medium for 48 h to establish TAMs (**Figure 4A**). We found that TAMs showed upregulation of M1 (CD86 and CD80) and M2 (CD163 and CD206) markers (**Figure S3B**). Then, TAMs were cultured in fresh medium for another 24 h, and ELISA assay showed that both TNF-α and IL-10 levels in the culture medium were significantly increased (**Figure S3C**), suggesting that CRC cells induce TAMs with a mixed M1/M2 phenotype, and these results are consistent with a previous study 9. After co-culturing with TAMs, as indicated in **Figure 4A**, HCT116 cells exhibited enhanced migratory capabilities (**Figure 4B**), with increased expressions of E-cadherin and vimentin, and decreased expression of N-cadherin (**Figure 4C**). Moreover, the representative markers of SPP1^+^TAMs such as SPP1, VEGFA and MIF were significantly increased, suggesting that SPP1^+^TAMs can be effectively simulated in the co-culture system and become activated when cells are exposed to CRC cell conditioned medium (**Figure 4D-E**). In addition, secreted levels of MIF were significantly higher in the conditioned medium of HCT-116 and LOVO cells when compared to the normal intestinal epithelial cells NCM460 (**Figure 4F**). We inferred that SPP1^+^TAMs not only as receivers of MIF signaling but also as important mediators and secondary senders (**Figure 1K**). Consistently, MIF increased in both tumor cells (**Figure 4F**) and activated SPP1^+^TAMs (**Figure 4G**). Therefore, during SPP1^+^TAMs activation, MIF is also secreted by SPP1^+^TAMs themselves, which may further increase SPP1^+^TAM activation.

We next used 4-Iodo-6-phenylpyrimidine (4-IPP), a suicide inhibitor of MIF 30 to further investigate the potential role of MIF in regulating SPP1^+^TAMs activation. As shown in Figure 4H, co-culture with TAMs increased HCT116 and LOVO cell migration, which was significantly reduced by 4-IPP in the co-culture system (**Figure 4H**). Meanwhile, 4-IPP significantly suppressed M2 macrophage activation as indicated by reduced CD206 expression and IL-10 secretion. However, 4-IPP did not affect expressions of M1 macrophage markers, such as CD86 and TNF-α) (**Figure S3D-E**). Additionally, 4-IPP also significantly reduced the expression levels of SPP1^+^TAMs markers in both HCT116-TAM and LOVO-TAM coculture systems (**Figure 4I**). These results suggest that MIF inhibitor 4-IPP blocks the MIF-mediated signaling in the CRC-TAM coculture system. Analysis of the CRC and SPP1^+^TAMs interactions highlighted three MIF-interacting receptors, they were CD44, CD74 and CXCR4, which were predicted to interact with MIF (**Figure 1I**). Further investigations found that CD44 expression was upregulated in TAMs, and reversed by MIF inhibitor 4-IPP (**Figure 4J**). We further investigated the roles of these three receptors in the activation of SPP1^+^TAMs. siRNA knockdown experiments showed that CD44 silencing more strongly reduced VEGF and MIF protein levels than CD74 or CXCR4 knockdown (**Figure S4**), suggesting that CD44 plays a crucial role in MIF-mediated angiogenesis during SPP1^+^TAM activation. scRNA-seq of mononuclear macrophage data from GSE164522 also confirmed the colocalization of SPP1 and CD44 (**Figure 4K-L**). These results suggest that CD44, rather than CD74 or CXCR4, is the primary mediator of SPP1^+^TAM activation.

### RNA-seq and ATAC-seq reveal an angiogenesis-related regulatory network involved in TAM activation

**Figure 5:**
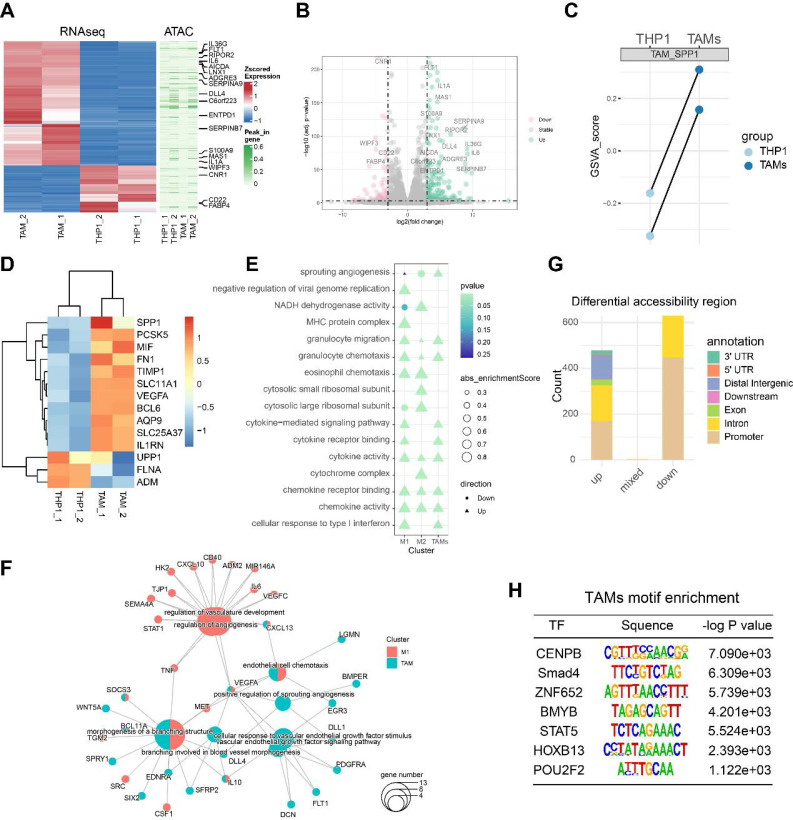
** RNA-seq and ATAC-seq revealed a transcription network potentially involved in TAM-mediated angiogenesis. (A)** Heatmap showing the differentially expressed genes (DEGs) between PMA-induced THP1 (M0 macrophages) and TAMs identified by RNA-seq; and the differentially accessible peaks between PMA-induced THP1 (M0 macrophages) and TAMs identified by ATAC-seq. **(B)** Volcano plots showing the DEGs between PMA-induced THP1 and TAMs identified by RNA-seq. **(C)** Paired dotplot showing the GSVA score of SPP1^+^TAMs markers in PMA-induced THP1 before and after TAMs activation.** (D)** The heatmap shows the expression levels of the SPP1^+^TAMs markers. **(E)** Comparison of the GO enrichment results of the DEGs between activated TAMs and M1/M2 macrophages. **(F)** The network diagram depicts the angiogenic pathway and the up-regulated genes that are enriched within this pathway. **(G)** Stacked bar plots show the number of the differentially accessible regions (DARs) identified from the ATAC-seq analysis. **(H)** Transcription factor binding motifs enriched in open chromatin regions of TAM, identified through Homer analysis.

Monocytes, macrophages and TAMs exhibit high plasticity within the immune microenvironment and undergo complex differentiation during cancer metastasis 6. Chromatin folding and transcription factor activation play important roles in the cellular differentiation process 31. To explore the transcriptomic and epigenomic changes during TAM differentiation, we performed RNA-seq and ATAC-seq on the PMA-induced THP1 (M0 macrophages) and TAMs. A cluster heatmap and PCA analysis of the RNA-seq data revealed clear between-group differences with minimal within-group variability (**Figure S5A-B**). After data preprocessing and quality control, we found that a total of 177 genes were significantly upregulated and 68 genes were downregulated, with |logFC| >3 and FDR < 0.001 as the thresholds (**Figure 5A-B**). The GSVA score of the SPP1^+^TAMs signature showed potent activation of SPP1^+^TAMs, suggesting a successful induction of SPP1^+^TAMs in our co-culture system (**Figure 5C-D**).

Next, we analyzed the THP-1 bulk RNA-seq dataset GSE154347, which contains transcriptome data of M0 macrophages and M1/M2 polarized macrophages. Differentially expressed genes (DEGs) were identified for M0 versus M1 and M0 versus M2. Based on the gene set enrichment analysis (GSEA), we observed an up-regulation of sprouting angiogenesis in both TAMs, and M1-polarized macrophage, with TAMs showing a higher enrichment score than M1 -polarized macrophage (**Figure 5E**). Next, we identified genes significantly upregulated in TAMs and M1-polarized macrophages, and performed over-representation enrichment analysis (ORA). Up-regulated genes in TAMs including VEGFA, WNT5A and IL10 were associated with angiogenesis during TAM activation; whereas STAT1, IL6 and TNF were associated with angiogenesis in M1-polarized macrophages (**Figure 5F**).

Quality control analysis of the ATAC-seq data revealed that fragment size distribution and library complexity were within a reasonable range (**Figure S5C**). PCA of the ATAC-seq data indicated that variability was primarily driven by intergroup differences (**Figure S5D**). Using Csaw 1.12.0 21 to quantify the ATAC signal intensity and identify differentially accessible regions (DARs), we detected a total of 478 significantly upregulated, and 929 significantly downregulated DARs (**Figure 5G**). These DARs were primarily located in the promoter region (**Figure S5E-F**). After peak calling, our enrichment analysis indicated that most peaks were centered around the promoter proximal to the transcription start site. Motif enrichment analysis revealed that the specific DNA binding of these transcription factors including POU2F2 and CENPB, were significantly associated with the open chromatin regions of TAMs (**Figure 5H**), suggesting that these transcription factors may contribute to TAM differentiation and warrant further validation.

### POU2F2 directly blinds to the promoter and activates BCL9L transcription, which is associated with MIF-driven SPP1^+^TAM activation

**Figure 6:**
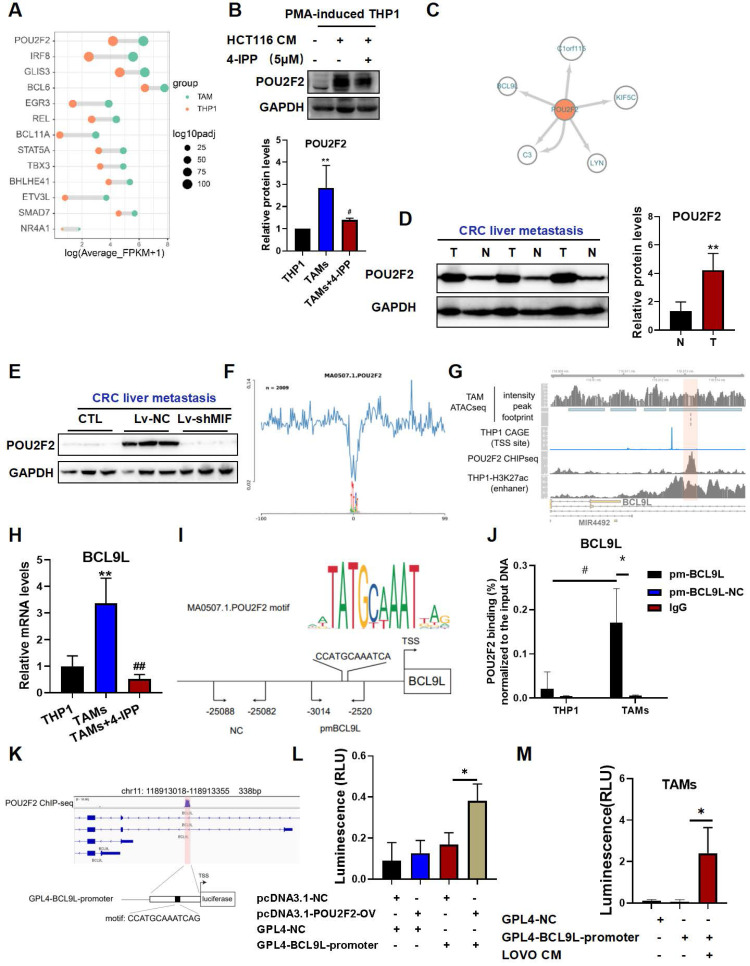
** POU2F2 directly binds to the promoter and activates BCL9L transcription, which is associated with MIF-regulated SPP1^+^TAM activation. (A)** The top activated transcription factors (TFs) in TAMs, ranked by adjusted *P*-value, as identified from RNA-seq data. **(B)** The protein levels of POU2F2 in THP-1 macrophages and CRC cells-TAMs co-culture system in the presence/absence of 4-IPP (upper panel); and the quantitative results were analyzed using Image J software (lower panel). Data are shown as Mean ± SD. *P<0.05, **P<0.01, *vs.* THP-1. ^#^*P*<0.05 *vs.* TAMs (n=3). **(C)** Regulatory networks of TFs associated with TAMs activation.** (D)** Protein levels of POU2F2 in the T and N of liver tissues in the CRC liver metastatic tissues were determined using the Western blotting (left panel); and the quantitative results were analyzed using Image J software (right panel). Data are shown as Mean ± SD. *P<0.05, *vs.* N (n=3). **(E)** Protein levels of POU2F2 in the livers with tumor of Lv-NC and shMIF-C group were determined using the Western blotting. **(F)** POU2F2 footprint in TAMs from the ATAC-seq data. **(G)** Track plot shows the footprint analysis results of the POU2F2 binding site, as well as the CAGE ChIP-seq results from the ENCODE database.** (H)** The mRNA levels of BCL9L in THP-1 macrophage and TAMs in the presence/absence of 4-IPP (left panel) were determined using the RT-qPCR analysis. Data are shown as Mean ± SD. **P<0.01, *vs.* THP-1; ^##^*P*<0.01, *vs.* TAMs (n=3). **(I)** JASPAR-predicted TF motif and schematic diagram showing the relative positions of qPCR probes for POU2F2-binding position validation in ChIP-qPCR assays.** (J)** Chromatin immunoprecipitated by the POU2F2 antibodies was analyzed by qPCR to assess POU2F2 binding. Data are shown as Mean ± SD. *P<0.05, *vs.* pm-BCL9L-NC; ^#^*P*<0.05, *vs.* THP1 (n=3). **(K)** Schematic diagram showing dual-luciferase reporter vector structure contain BCL9L promoter. **(L)** Dual-luciferase reporter assay showing the transcriptional activation effect of POU2F2 on BCL9L promoter sequences. Data are shown as Mean ± SD. *P<0.05, NC+BCL9L-promoter *vs.* POU2F2-OV+BCL9L-promoter (n=3).** (M)** Dual-luciferase reporter assay showing that the BCL9L promoter sequences were transcriptionally activated after TAMs activation. Data are shown as Mean ± SD. *P<0.05, GPL4-BCL9L-promoter *vs.* LOVO-CM + GPL4-BCL9L-promoter (n=3). N: adjacent normal tissues; T: tumor tissue; NC: negative control primer; pmBCL9L: BCL9L promoter primer.

We screened the significantly upregulated transcription factors from our RNA-seq data (**Figure 6A**), and intersected this set with the genes in the TAM-enriched motifs and transcription factor footprints. It was found that POU2F2 were not only enriched in the motif analysis but was also significantly upregulated at the transcriptional level. We further used the MIF inhibitor 4-IPP to screen for the MIF-regulated transcription factors. We found that POU2F2 was significantly upregulated in TAMs; and this effect was significantly attenuated by 4-IPP, suggesting that POU2F2 is regulated by MIF signaling (**Figure 6B**). Furthermore, we constructed a transcription factor regulatory network associated with TAM activation by using the ATAC-seq footprint analysis (**Figure 6C**). Consistent with a role in metastasis, POU2F2 expression was significantly upregulated at liver metastatic tumors compared with adjacent normal tissues (**Figure 6D**). Furthermore, POU2F2 expression was significantly reduced in shMIF-CT26 liver metastasis when compared with Lv-NC (**Figure 6E**), suggesting that POU2F2 is an essential transcription factor regulated by MIF. Given the role of POU2F2 in macrophages remains unclear, we explored its downstream targets. BCL9L was identified as a downstream target of POU2F2 with a high binding score (**Figure 6C and 6F**). Integration of POU2F2 ChIP-seq and CAGE-seq (cap analysis of gene expression and deep sequencing) data from ENCODE database (https://www.encodeproject.org/) revealed strong POU2F2 binding at the chr11:118913185-118913197 (hg38) region, which a THP-1 enhancer region near the transcription start site (TSS) of BCL9L (**Figure 6G**). The RT‒qPCR results also confirmed that BCL9L activation was significantly dependent on MIF signaling during TAM activation (**Figure 6H**).

To further validate the POU2F2 transcription binding site, we designed primers spanning the predicted binding sites for ChIP‒qPCR validation (**Figure 6I**). Results revealed a high enrichment at the BCL9L promoter region but not at the negative control region (**Figure 6J**). We constructed luciferase reporter vectors covering the specific region shown in **Figure 6K**, which is overlapped the POU2F2 ChIP-seq peak region and included the predicted POU2F2 motif site (**Figure 6K**). Dual-luciferase reporter assays showed significantly increased luminescence in the POU2F2-OV/GLP4-BCL9L-promoter co-transfection group (**Figure 6L**). Moreover, the CRC cell-TAM coculture system also significantly activated GLP4-BCL9L-promoter transcription (**Figure 6M**). These findings suggest that POU2F2 activates BCL9L transcription by specifically binding the BCL9L promoter.

### MIF drives pro-angiogenic SPP1^+^TAMs activation through the POU2F2/BCL9L signaling axis

**Figure 7:**
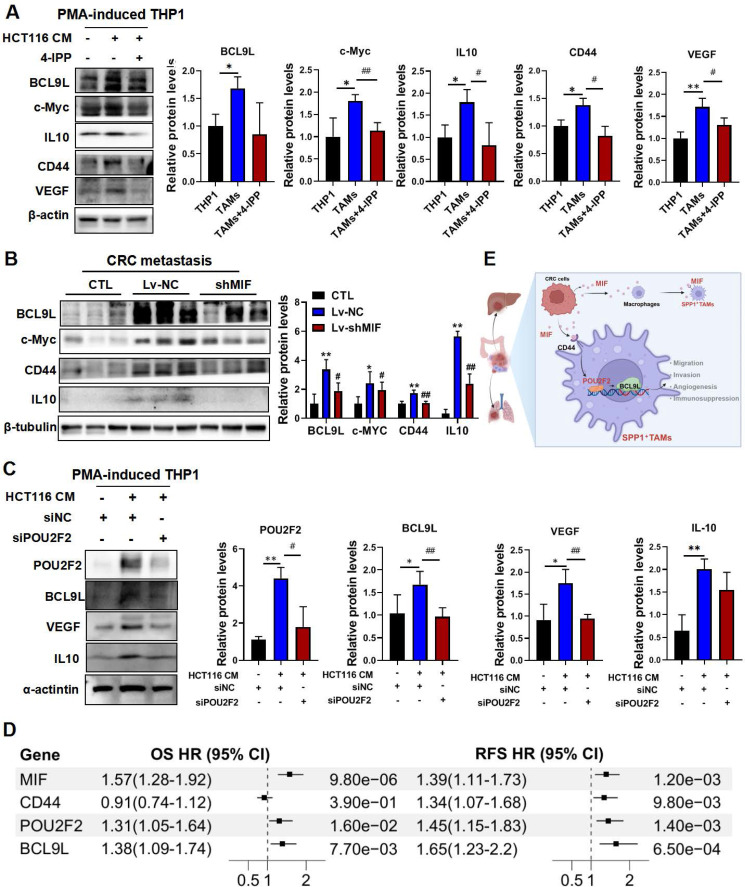
** MIF promotes the activation of proangiogenic SPP1^+^TAMs through POU2F2/BCL9L signaling. (A)** The protein levels of BCL9L and its downstream proteins including IL10, c-Myc, CD44 and VEGF in THP-1, TAM and TAM+4-IPP groups were determined by using Western blotting. Data are shown as Mean ± SD. *P<0.05, **P<0.01, *vs.* THP-1 cells; ^#^*P*<0.05, ^##^*P*<0.01, *vs.* TAMs (n=3). **(B)** The protein levels of BCL9L and its downstream proteins including IL10, c-Myc and CD44 in the lung tissues of control, Lv-NC and Lv-shMIF groups were determined by Western blotting. Data are shown as Mean ± SD. *P<0.05, **P<0.01, *vs.* CTL; ^#^*P*<0.05, ^##^*P*<0.01, *vs.* Lv-NC (n=3). **(C)** The protein levels of POU2F2 downstream proteins including BCL9L, POU2F2, VEGF and IL10 in CRC cell-TAM coculture model after POU2F2 knockdown. Data are shown as Mean ± SD. *P<0.05, **P<0.01, *vs.* HCT116-CM; ^#^*P*<0.05, ^##^*P*<0.01, *vs.* siNC (n=3). **(D)** The forest plot showing the univariate Cox regression analyses of the MIF/CD44/POU2F2/BCL9L axis for OS and RFS based on integrated CRC cohorts.** (E)** The diagram shows MIF activated the SPP1^+^TAMs mediated proangiogenic phenotype through the MIF/POU2F2/BCL9L axis and ultimately enhanced CRC metastasis. OS, overall survival; RFS, recurrence-free survival; CI, confidence interval.

The Wnt signaling pathway is closely associated with cancer progression. BCL9L and BCL9 are essential β-catenin coactivators within this pathway 32, which have also been identified as potential therapeutic targets for CRC 33. Moreover, studies have shown that BCL9/β-catenin increases the expression of downstream genes such as CD44, IL10 and VEGF^33^, which are involved in SPP1^+^TAMs activation. Importantly, VEGF is recognized as a marker of SPP1^+^TAMs and is a key proangiogenic factor 4. Here, we found that VEGF may be regulated by MIF/POU2F2/BCL9L. BCL9L and its downstream targets including IL10, c-Myc, CD44 and VEGF were indeed activated in CRC-TAM co-culture system, and these effects were significantly inhibited by 4-IPP (**Figure 7A**). We further validated these downstream targets in the lung metastasis tissue samples from MIF-knockdown mice (**Figure 7B**). Additionally, silencing POU2F2 with siPOU2F2 confirmed its regulatory role in the expressions of BCL9L, VEGF and IL10 (**Figure 7C**). We integrated the public CRC cohort and performed univariate Cox regression analyses to assess the clinical correlations of these core targets. Elevated expressions of MIF, CD44, POU2F2 and BCL9L within the MIF signaling axis were associated with poor prognosis, evidenced by a strong association with overall survival and recurrence-free survival (RFS) (**Figure 7D**). These findings collectively suggest that the proangiogenic phenotype of SPP1^+^TAMs is activated by the MIF/POU2F2/BCL9L axis (**Figure 7E**).

## Discussion

In most solid tumors, macrophages infiltration correlates with poor prognosis. However, in CRC, studies report conflicting associations, with some linking TAMs infiltration to improved prognosis 34, 35. These conflicting results may be attributed to the co-existence of both M1 and M2 macrophages, as well as the cellular heterogeneity of TAMs within tumors. TAMs display hybrid phenotypes within the complex TME. Here, our established CRC-TAM coculture model also showed a mixed M1/M2 phenotype, which aligns with previous findings 9. Spatial heterogeneity may also contribute to these conflicting results. TAMs located at the invasive front of tumors often exhibit antitumor activity, whereas those in the tumor center exhibit protumor properties. These observations highlight the limitations of the binary M1/M2 framework 6 and the oversimplification of macrophage plasticity in the complex TME 36. It is crucial to further investigate the granular phenotypic and spatial subtyping of macrophages in the TME.

TAMs within the TME comprise both monocyte-derived and tissue-resident populations, with recruited monocytes often predominating during metastasis 37. Because PMA-differentiated THP-1-derived macrophages originate from a monocytic lineage, they model a major subset of human TAMs. Accordingly, we used PMA-differentiated THP-1 macrophages *in vitro* to investigate the mechanistic role of the MIF/CD44 axis in TAM activation. We used CRC cell-conditioned medium to mimic the paracrine, unidirectional influence of tumor cells on macrophages while minimizing confounding signals from other immune cells, which enabled us to analyze the CRC-TAM interactions that drive tumor progression and metastasis. To further, we will validate these findings by isolating primary human peripheral blood monocytes and differentiate them into macrophages using GM-CSF. Using both CRC-TAM co-culture and CRC cell-conditioned medium-induced TAM activation, we will explore TAM heterogeneity to facilitate clinical translation.

Recent studies have identified distinct macrophage clusters with unique transcriptomic and proteomic features 38. Notably, scRNA-seq has gained attraction for defining the TME and revealing its cellular heterogeneity. A recent study has demonstrated that CXCL9:SPP1 macrophage polarity has emerged as a strong prognostic indicator in the TME across multiple types of cancers 8. SPP1 is a marker gene for SPP1^+^TAMs, correlates closely with tumor metastasis and prognosis 5. Therefore, compared with the traditional M1/M2 classification, using SPP1 as a marker to monitor therapeutic efficacy may provide superior prognostic value. Consequently, it is critical to identify CRC TAM subpopulations that drive tumor promotion and metastasis and to develop TAM-targeted therapeutic strategies.

SPP1^+^TAMs, which are characterized by a proangiogenic and tumorigenic phenotype, were initially identified from a subpopulation of macrophages in CRC samples 4. However, these cells exhibit resistance to current TAM-targeted therapies 4. Specific interventions against SPP1**^+^** TAMs remain unavailable. Therefore, this study aimed to identify targets to suppress SPP1**^+^** TAMs and to investigate themechanisms underlying SPP1**^+^** TAM activation both *in vitro* and *in vivo*.

MIF is one of the earliest identified functional cytokines, its elevated expression significantly impacts many physiological and pathological processes, including cancer and inflammatory diseases 11. In ulcerative colitis (UC), inflammation severity correlates with MIF expression and secretion 39. MIF may play a pivotal role in the early inflammation-to-cancer transition and CRC-associated carcinogenesis 40. Inhibiting MIF could be important for preventing the progression from UC to CRC.

Here, our cell‒cell interaction analysis revealed a potential link between tumor-derived MIF and SPP1^+^TAMs activation. MIF also promotes angiogenesis under hypoxic conditions, which is a hallmark of malignancy and a promising target for therapeutic intervention 41. Macrophages contribute to promote angiogenesis during cancer progression and metastasis 42. Studies have demonstrated that MIF expression is upregulated under hypoxic and low glucose conditions, both of which are typical activators of angiogenesis 13. Under hypoxic conditions, HIF-1α can significantly upregulates MIF expression 43, and interactions between MIF and HIF-1α has been observed during tumorigenesis 44. Taken together, these findings indicate that MIF is potentially associated with angiogenesis in the cancer development, although the underlying mechanism remains unclear.

In this study, we revealed that MIF is the most significant signaling interaction between CRC cells and SPP1^+^TAMs. Moreover, 4-IPP, a MIF inhibitor, significantly reduced the expressions of SPP1^+^TAMs-associated markers (**Figure 4I**). We also utilized lentiviral transfection to establish a MIF knockdown model for CRC liver metastasis and CRC lung metastasis mouse models. Subsequent *in vivo* experiments revealed a significant reduction in the metastatic potential of CRC cells (**Figure 3**). MIF knockdown animal models underscore its important role in the communication between CRC cells and TAMs. Moreover, immunofluorescence co-localization analysis and Western blotting further confirmed a notable reduction in the abundance of SPP1^+^TAMs within the tumor. These findings suggest that MIF is a critical regulator of proangiogenic SPP1^+^TAMs.

The three-dimensional structure of chromatin is critical for regulating the open and closed states of gene promoters and enhancers, thereby influencing gene expression, cellular characteristics, and biological development. This structural configuration is closely associated with developmental abnormalities, human diseases including cancer progression 31. The state of chromatin determines fundamental cellular processes, such as gene expression and DNA replication. Different open chromatin regions contain distinct regulatory sequences, including promoters, enhancers, insulators, and locus control regions. These sequences interact with cell type-specific transcription factors to execute transcriptional programs that guide cellular differentiation and development 45. To identify key transcription factors related to TAM differentiation, we performed the RNA-seq and ATAC-seq on the TAMs before and after differentiation. We observed significant changes in the transcriptome and epigenome related to angiogenesis during the TAM differentiation process. Next, we used GSEA to compare the DEGs across TAMs and M1/M2 polarized macrophages. GO enrichment results indicate that M1 polarization and TAMs activation are functionally associated with angiogenesis, with a higher enrichment score observed in TAMs (**Figure 5E**). Similar results were observed in another study, the secretion of FABP4 by M1-polarized macrophages promotes angiogenesis and exacerbates inflammatory disease 46.

By integrating RNA-seq data, transcription factor footprint analysis, and ATAC-seq motif enrichment, we identified potential transcription factors implicated in TAM activation, including POU2F2 and STAT5 (**Figure 5H**). To determine which are regulated by MIF, we conducted a comparative functional assessment *in vitro*. Results revealed that POU2F2 is the predominant transcription factor that is regulated by MIF (**Figure 6B**). Transcription factor footprint and ChIP-qPCR further indicated that POU2F2 engages the promoters of BCL9L and C3, with MIF showing a stronger regulator effect on BCL9L.

BCL9L and its homologous gene B-cell lymphoma 9 (BCL9) share similar structures and function as important transcriptional co-activators and positive regulators within the β-catenin transcriptional activation complex of the Wnt signaling pathway. Previous studies have shown that targeting BCL9/β-catenin can effectively inhibits CRC tumor growth 33. Activation of the BCL9L/β-catenin complex upregulates VEGF and CD44 in multiple myeloma and CRC tissues 32, potentially aligning with the SPP1^+^TAMs activation observed in our study. We found that CD44 and VEGF are regulated by the MIF/POU2F2/BCL9L axis and contribute to the proangiogenic SPP1^+^TAMs activation.

Reprogramming TAMs within TME has emerged as a pivotal strategy for TAM-targeted therapy and drug development 6. Several drugs targeting TAMs have progressed to phase I and II clinical trials, including inhibitors that blocks macrophages recruitment, such as CSF1R; and the agent that suppresses macrophages activation, such as CD40 6. Although macrophage-targeted therapies offer unique advantages in treating tumor metastasis, it also encounters limitations and challenges in clinical translation. A key challenge is selecting the most appropriate target and TAM subpopulation, particularly for SPP1^+^TAM-targeted intervention. Here, we have demonstrated that MIF signaling regulates POU2F2/BCL9L and their downstream effectors.

In addition, we systematically compared the functional roles of MIF receptor in TAMs activation. CD44 showed the strongest regulatory relationship and was suppressed by MIF inhibitors (**Figure 4J**). Meanwhile, we also found that CD44 and CD74 expressions varied across different stimulation conditions (**Figure 4J**, **Figure S4**). Although CD74 indeed showed an upward trend, the change in CD44 expression was markedly more pronounced. By contrast, CXCR4 expression did not different between the two culture systems. Collectively, the stimulatory effect of this tumor-conditioned medium on CD44 expression was significantly stronger than that on CD74 or CXCR4. More importantly, knockdown of CD44 markedly reduced SPP1 and VEGF expressions, whereas knockdown of CD74 or CXCR4 had no significant effect on either factors. These results strongly support that MIF mediates its downstream functions primarily through CD44 (**Figure S4**).

CD44 is a complex transmembrane glycoprotein involved in adhesion, migration, proliferation, apoptosis and angiogenesis, and it serves as a tumor stem cell marker, these functions are closely associated with tumor malignancy 47. Nevertheless, roles of CD44 in TAMs remain underexplored. Recent scRNA-seq studies have started to elucidate the functions associated with CD44 in TAMs, revealing a strong correlation between CD44-positive macrophages and angiogenic activity 48. Nonetheless, the macrophage-specific functions of CD44 remain poorly understood. Here, we delineated a MIF-CD44-POU2F2/BCL9L signaling axis in TAMs, whereby CD44 activation functions as a key amplifier of MIF signaling. Targeting MIF or CD44 presents a novel strategy. Development of receptor-targeted agents, including monoclonal antibodies against CD44, is a realistic therapeutic avenue 49.

## Conclusions

In summary, we identify POU2F2 as a crucial transcription factor that drives SPP1^+^TAMs activation by directly regulating BCL9L transcription. MIF promotes the proangiogenic phenotype of SPP1^+^TAMs* via* the CD44/POU2F2/BCL9L axis, thereby enhancing CRC metastasis. Furthermore, CD44 (a MIF receptor) is co-expressed with SPP1, and CD44 is essential for MIF-mediated angiogenesis in SPP1^+^TAMs. Therefore, targeting this novel axis could effectively suppress SPP1^+^TAMs activation and represents a promising strategy for managing CRC metastasis.

## Supplementary Material

Supplementary figures and tables.

## Data Availability

The RNA-seq and ATAC-seq FASTQ files from this study are available in the CNCB database (https://www.cncb.ac.cn/) under accession numbers HRA007197 and HRA007209 respectively.

## Abbreviations

ATAC-seq: Assay for Transposase Accessible Chromatin with high-throughput sequencing
cDNA: complementary DNA
BCA: Bicinchoninic acid assay
ChIP-seq: Chromatin Immunoprecipitation Sequencing
CI: confidence interval
CRC: Colorectal cancer
COAD: Colon cancer cohort from TCGA
READ: Rectal Cancer cohort from TCGA
CRLM: Colorectal cancer liver metastasis
DAR: Differential accessible region
DEGs: Differentially expressed genes
DMSO: Dimethyl sulfoxide
E-cad: E-cadherin
ELISA: Enzyme-linked immunosorbent assay
EMT: Epithelial-mesenchymal transition
GFP: Green fluorescent protein
GSEA: Gene Set Enrichment Analysis
GO: Gene Ontolog
HUVECs: human umbilical vein endothelial cells
KEGG: Kyoto Encyclopedia of Genes and Genomes
MIF: Macrophage migration inhibitory factor
N-cad: N-cadherin
ORA: Over representation analysis
OS: overall survival
PCA: Principal component analysis
RFS: recurrence-free survival
RNA-seq: RNA sequencing
PMA: Phorbol 12-myristate 13-acetate
TCGA: The Cancer Genome Atlas
TAMs: Tumor-associated macrophages
TME: Tumor microenvironment
UMAP: Uniform Manifold Approximation and Projection for Dimension Reduction
4-IPP: 4-Iodo-6-phenylpyrimidine
