# Supplementary material for: CD44/POU2F2/BCL9L axis mediates MIF-driven SPP1+TAM activation in colorectal cancer metastasis
Source: Int J Biol Sci. 2026 Feb 4;22(5):2492–511. doi: 10.7150/ijbs.116575 (PMC12965218; doi:10.7150/ijbs.116575)
Supplement: Supplementary file 1 — Supplementary figures and tables. [file ijbsv22p2492s1.pdf]

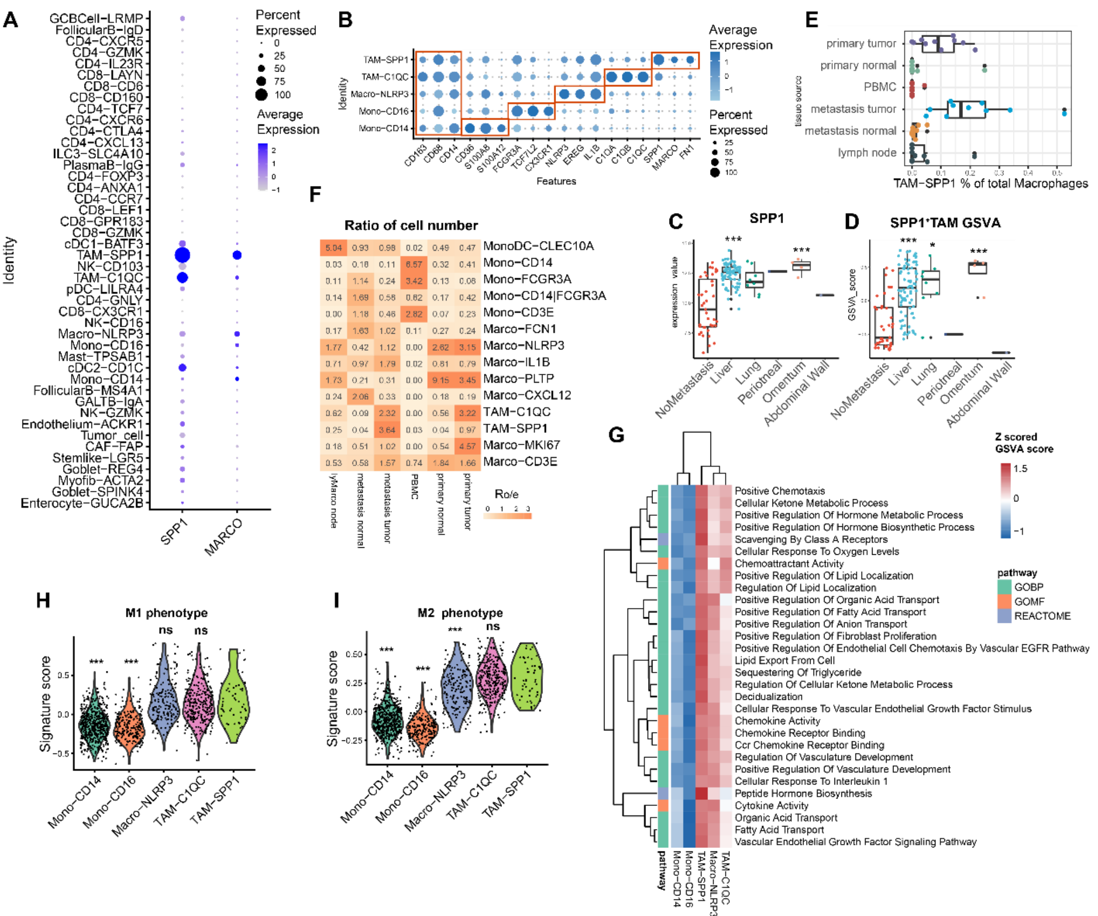

Supplementary Figure S1. Characteristics of SPP1⁺TAMs identified from the CRC scRNA-seq data.

(A) SPP1 and MARCO expression value compared across cell type in GSE146771. (B) Dotplot displaying the expression of myeloid marker and monocyte and macrophage sub-clusters marker. **(C-D)** The boxplot shows the expression of SPP1 (C) and SPP1⁺TAMs GSVA score **(D)** in non-metastatic CRC and different types of CRC metastasis tissues. **P*＜0.05, ***P*＜0.01, ****P*<0.001 *vs.* No metastasis. **(E)** The percentage of SPP1⁺TAMs among the total macrophages in different tumor tissues based on scRNA-seq data from CRC liver metastatic tissues. **(F)** The tissue distribution ratio of each monocyte and macrophage cluster estimated by the Ro/e score in CRC liver metastases. **(G)** Heatmap showing the results of the GSVA enrichment analysis of monocyte and macrophage population subsets, highlighting the pathways that were significantly upregulated in the SPP1⁺TAMs. **(H-I)** M1 and M2 signature were compared across multiple monocyte and macrophage clusters. *ns*, not significant; ****P*<0.001 *vs.* TAM-SPP1.

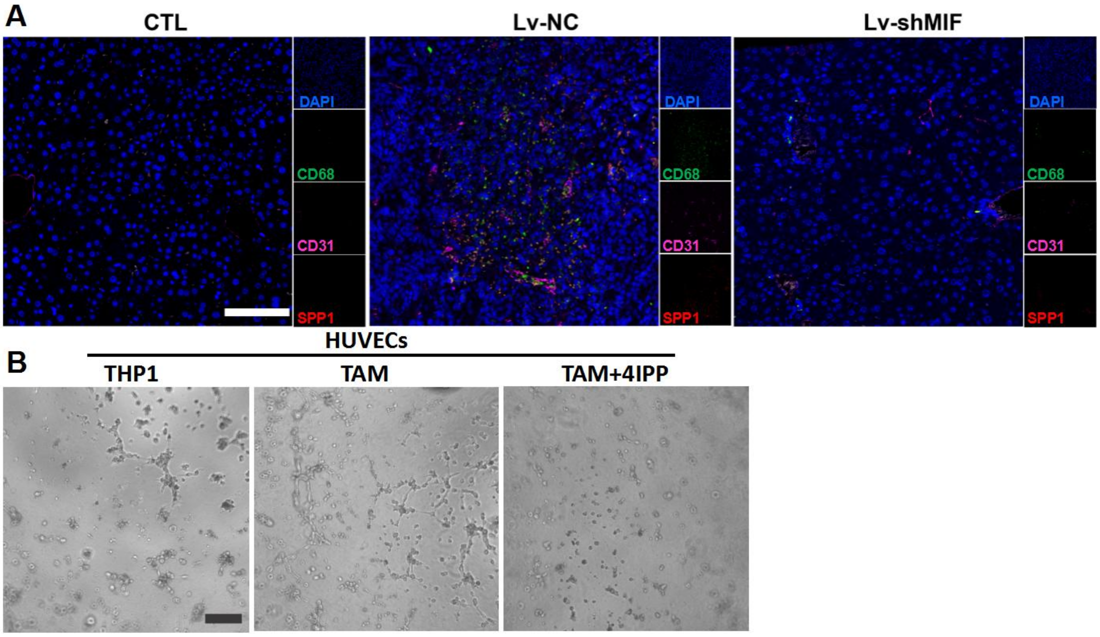

Supplementary Figure S2. MIF is essential for the activation of SPP1^{+}TAM-mediated angiogenesis. (A) Representative immunofluorescence images of CRC liver metastasis model showing the abundance of SPP1^{+}TAMs and its co-localization of the microvessels (scale bar =100 μm). CTL: control mice; Lv-NC: CT26 liver metastasis model mice; Lv-shMIF: CT26 liver metastasis model with MIF knockdown. (B) Effect of the condition median from THP1, TAM and 4-IPP treated TAMs on tube formation ability of HUVECs by tube formation assay (scale bar =100 μm).

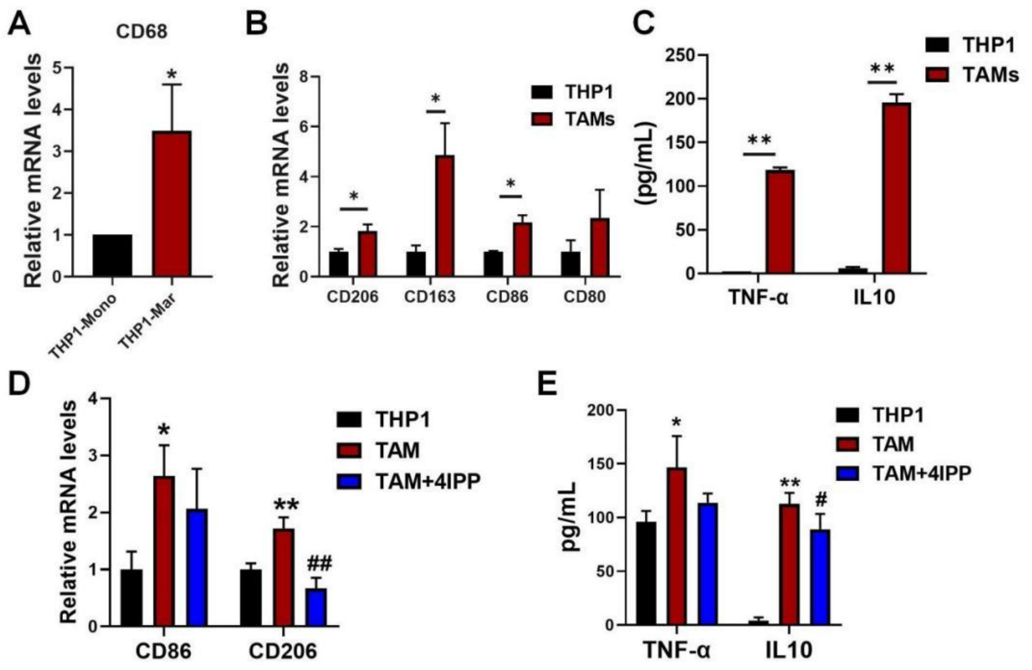

Supplementary Figure S3. Characterization of PMA-induced THP-1 macrophages and their M1/M2 polarization phenotype in co-culture model.

(A) The mRNA levels of CD68 were determined using the RT–qPCR. PMA was used to induce the differentiation of THP-1 cells into macrophages for 24 h. Data are shown as Mean ± SD. *$P<$ 0.05, *vs.*THP-1-Mono group (n=3). **(B)** The mRNA levels of the M1 macrophage markers (CD86, CD80), and the M2 macrophage markers (CD206 and CD163) in PMA-induced THP-1 macrophages or TAMs were determined by using the RT–qPCR assay (n=3). **(C)** The levels of TNF-α and IL10 in PMA-induced THP-1 macrophages or TAM were determined using the ELISA assay. Data are shown as Mean ± SD. **$P<0.01$ *vs.* THP-1 group (n=3). **(D)** The mRNA levels of the M1 macrophage markers (CD86) and the M2 macrophage markers (CD206) in PMA-induced THP-1 macrophages, TAMs or 4-IPP treated TAMs were measured by RT–qPCR (n=3). **(E)** The levels of TNF-α and IL10 in PMA-induced THP-1 macrophages, TAMs or 4-IPP treated TAMs were measured by ELISA assay. *$P<0.05$, **$P<0.01$, *vs.* THP-1; $^{\#}P<0.05$, $^{\#\#}P<0.01$, *vs.* TAMs (n=3).

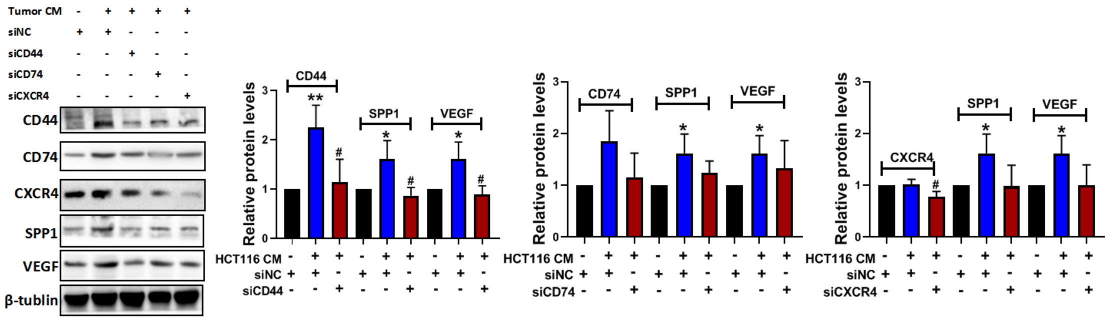

Supplementary Figure S4. Effects of the three receptors on the activation of SPP1$^+$ TAMs determined by siRNA knockdown assays. The protein expression levels of SPP1$^+$TAM markers in TAM cells after siRNA-mediated knockdown of MIF receptors (CD44, CD74 and CXCR4) were determined by Western blotting (left panel), and quantified data were shown using Image J software (right panel). Data are shown as Mean ± SD (n=3). $^{*}P<0.05$, *vs.* the corresponding control; $^{\#}P<0.05$ *vs.* si-NC.

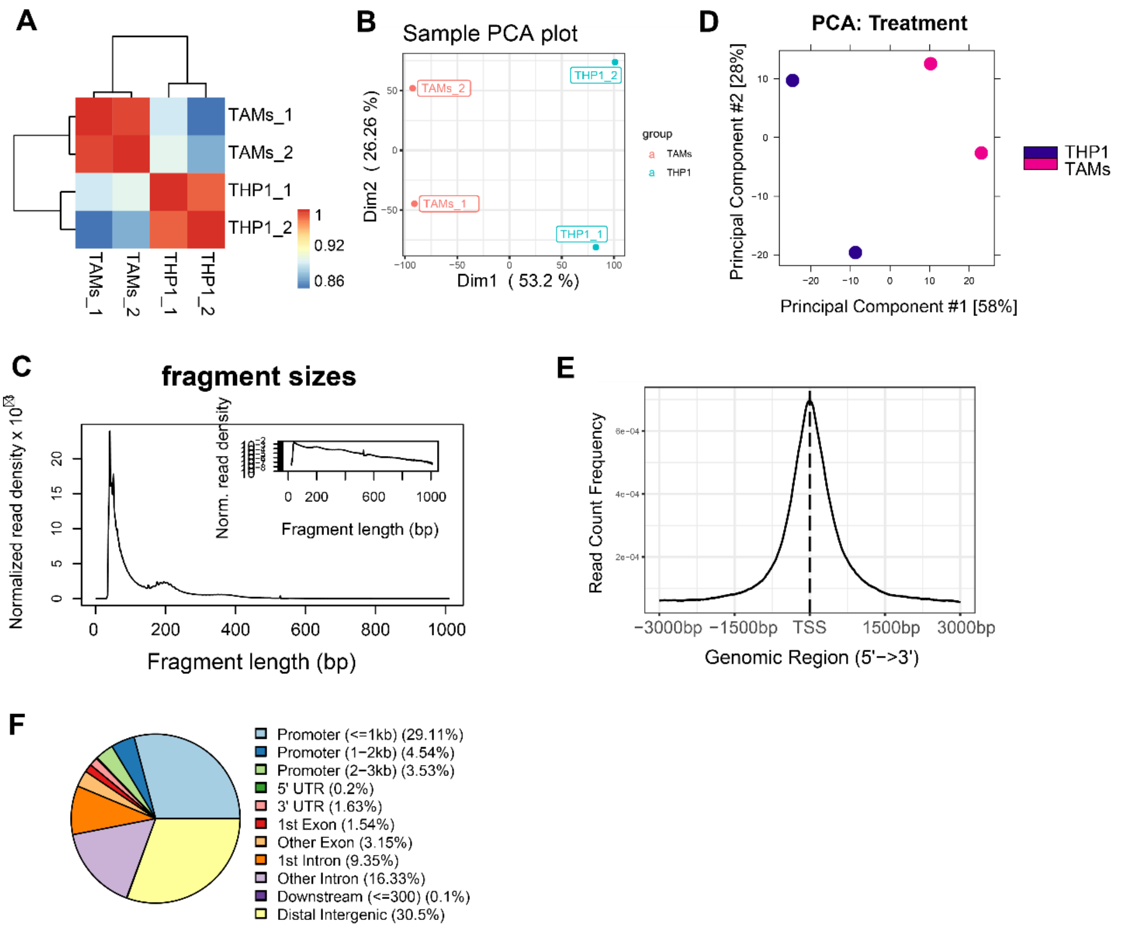

Supplementary Figure S5. Quality control for the TAMs RNA-seq and ATAC-seq data. **(A-B)** Cluster heatmap **(A)** and PCA plot **(B)** showing the correlation of RNA-seq data between two group samples. **(C)** Fragment size distribution of ATAC-seq reads. **(D)** PCA plot showing the correlation of ATAC-seq data between two groups of samples. **(E)** Average ATAC-seq peak intensity near the transcription start site (TSS). **(F)** The pie plot displays the genomic annotation of ATAC-seq peak locations.

Supplementary Table S1. The signaling pathways involved in the interaction between tumor cells and the SPP1^{+}TAMs.

source	target	ligand	receptor	prob	pval	interaction_name	interaction_name_2	pathway_name	annotation	evidence
Tumor_cell	TAM-SPP1	MIF	CD74_CD44	0.353098	0	MIF_CD74_CD44	MIF - (CD74+CD44)	MIF	Secreted Signaling	PMID: 29637711; PMID: 26175090
Tumor_cell	TAM-SPP1	MIF	CD74_CXCR4	0.304816	0	MIF_CD74_CXCR4	MIF - (CD74+CXCR4)	MIF	Secreted Signaling	PMID: 29637711; PMID: 24760155
Tumor_cell	TAM-SPP1	LAMB3	CD44	0.237938	0	LAMB3_CD44	LAMB3 - CD44	LAMININ	ECM-Receptor	KEGG: hsa04512
Tumor_cell	TAM-SPP1	MDK	NCL	0.216687	0	MDK_NCL	MDK - NCL	MK	Secreted Signaling	PMID: 28356350
Tumor_cell	TAM-SPP1	APP	CD74	0.209625	0	APP_CD74	APP - CD74	APP	Cell-Cell Contact	PMID: 19849849
Tumor_cell	TAM-SPP1	LAMC2	CD44	0.202936	0	LAMC2_CD44	LAMC2 - CD44	LAMININ	ECM-Receptor	KEGG: hsa04512
Tumor_cell	TAM-SPP1	LAMB1	CD44	0.152977	0	LAMB1_CD44	LAMB1 - CD44	LAMININ	ECM-Receptor	KEGG: hsa04512
Tumor_cell	TAM-SPP1	MDK	SDC4	0.13288	0	MDK_SDC4	MDK - SDC4	MK	Secreted Signaling	PMID: 28356350
Tumor_cell	TAM-SPP1	LAMA3	CD44	0.129044	0	LAMA3_CD44	LAMA3 - CD44	LAMININ	ECM-Receptor	KEGG: hsa04512
Tumor_cell	TAM-SPP1	LAMA5	CD44	0.108809	0	LAMA5_CD44	LAMA5 - CD44	LAMININ	ECM-Receptor	KEGG: hsa04512
Tumor_cell	TAM-SPP1	NAMPT	ITGA5_ITGB1	0.106967	0.02	NAMPT_ITGA5_ITGB1	NAMPT - (ITGA5+ITGB1)	VISFATIN	Secreted Signaling	PMID: 28490838

Tumor_cell	TAM-SPP1	LAMC1	CD44	0.105072	0	LAMC1_CD44	LAMC1 - CD44	LAMININ	ECM-Receptor	KEGG: hsa04512
Tumor_cell	TAM-SPP1	MDK	LRP1	0.090575	0	MDK_LRP1	MDK - LRP1	MK	Secreted Signaling	PMID: 28356350
Tumor_cell	TAM-SPP1	LAMB2	CD44	0.079177	0	LAMB2_CD44	LAMB2 - CD44	LAMININ	ECM-Receptor	KEGG: hsa04512
Tumor_cell	TAM-SPP1	F11R	ITGAL_ITGB2	0.068683	0	JAM1_ITGAL_ITGB2	JAM1 - (ITGAL+ITGB2)	JAM	Cell-Cell Contact	PMID: 11812992
Tumor_cell	TAM-SPP1	MDK	SDC2	0.053338	0	MDK_SDC2	MDK - SDC2	MK	Secreted Signaling	PMID: 28356350
Tumor_cell	TAM-SPP1	MDK	ITGA4_ITGB1	0.050509	0	MDK_ITGA4_ITGB1	MDK - (ITGA4+ITGB1)	MK	Secreted Signaling	PMID: 28356350
Tumor_cell	TAM-SPP1	PTPRC	MRC1	0.041412	0.01	PTPRC_MRC1	PTPRC - MRC1	CD45	Cell-Cell Contact	PMID: 27601670; PMID: 10575006
Tumor_cell	TAM-SPP1	LAMB3	ITGA3_ITGB1	0.036748	0	LAMB3_ITGA3_ITGB1	LAMB3 - (ITGA3+ITGB1)	LAMININ	ECM-Receptor	KEGG: hsa04512
Tumor_cell	TAM-SPP1	VEGFA	FLT1	0.032212	0	VEGFA_VEGFR1	VEGFA - VEGFR1	VEGF	Secreted Signaling	KEGG: hsa04370; PMID: 16633338
Tumor_cell	TAM-SPP1	LAMB3	ITGA1_ITGB1	0.031835	0	LAMB3_ITGA1_ITGB1	LAMB3 - (ITGA1+ITGB1)	LAMININ	ECM-Receptor	KEGG: hsa04512
Tumor_cell	TAM-SPP1	LAMC2	ITGA3_ITGB1	0.03017	0	LAMC2_ITGA3_ITGB1	LAMC2 - (ITGA3+ITGB1)	LAMININ	ECM-Receptor	KEGG: hsa04512
Tumor_cell	TAM-SPP1	THBS1	SDC4	0.029995	0	THBS1_SDC4	THBS1 - SDC4	THBS	ECM-Receptor	KEGG: hsa04512
Tumor_cell	TAM-SPP1	LAMC2	ITGA1_ITGB1	0.026113	0	LAMC2_ITGA1_ITGB1	LAMC2 - (ITGA1+ITGB1)	LAMININ	ECM-Receptor	KEGG: hsa04512

Tumor_cell	TAM-SPP1	FN1	CD44	0.025021	0	FN1_CD44	FN1 - CD44	FN1	ECM-Receptor	KEGG: hsa04512
Tumor_cell	TAM-SPP1	COL9A3	CD44	0.023365	0	COL9A3_CD44	COL9A3 - CD44	COLLAGEN	ECM-Receptor	KEGG: hsa04512
Tumor_cell	TAM-SPP1	EFNA1	EPHA1	0.021593	0	EFNA1_EPHA1	EFNA1 - EPHA1	EPHA	Cell-Cell Contact	PMID: 15114347
Tumor_cell	TAM-SPP1	LAMB1	ITGA3_ITGB1	0.021591	0	LAMB1_ITGA3_ITGB1	LAMB1 - (ITGA3+ITGB1)	LAMININ	ECM-Receptor	KEGG: hsa04512
Tumor_cell	TAM-SPP1	IL7	IL7R_IL2RG	0.019014	0	IL7_IL7R_IL2RG	IL7 - (IL7R+IL2RG)	IL2	Secreted Signaling	KEGG: hsa04060
Tumor_cell	TAM-SPP1	LAMB1	ITGA1_ITGB1	0.018665	0	LAMB1_ITGA1_ITGB1	LAMB1 - (ITGA1+ITGB1)	LAMININ	ECM-Receptor	KEGG: hsa04512
Tumor_cell	TAM-SPP1	SCGB3A2	MARCO	0.018417	0	SCGB3A2_MARCO	SCGB3A2 - MARCO	UGRP1	Secreted Signaling	PMID: 12847263
Tumor_cell	TAM-SPP1	LAMA3	ITGA3_ITGB1	0.017782	0	LAMA3_ITGA3_ITGB1	LAMA3 - (ITGA3+ITGB1)	LAMININ	ECM-Receptor	KEGG: hsa04512
Tumor_cell	TAM-SPP1	CDH1	CDH1	0.015384	0	CDH1_CDH1	CDH1 - CDH1	CDH	Cell-Cell Contact	KEGG: hsa04514
Tumor_cell	TAM-SPP1	LAMA3	ITGA1_ITGB1	0.015364	0	LAMA3_ITGA1_ITGB1	LAMA3 - (ITGA1+ITGB1)	LAMININ	ECM-Receptor	KEGG: hsa04512
Tumor_cell	TAM-SPP1	F11R	F11R	0.01524	0	F11R_F11R	F11R - F11R	JAM	Cell-Cell Contact	KEGG: hsa04514
Tumor_cell	TAM-SPP1	SEMA3C	NRP1_NRP2	0.014757	0	SEMA3C_NRP1_NRP2	SEMA3C - (NRP1+NRP2)	SEMA3	Secreted Signaling	PMID: 27533782
Tumor_cell	TAM-SPP1	LAMA5	ITGA3_ITGB1	0.014699	0	LAMA5_ITGA3_ITGB1	LAMA5 - (ITGA3+ITGB1)	LAMININ	ECM-Receptor	KEGG: hsa04512

Tumor_cell	TAM-SPP1	LAMC1	ITGA3_ITGB1	0.014143	0	LAMC1_ITGA3_ITGB1	LAMC1 - (ITGA3+ITGB1)	LAMININ	ECM-Receptor	KEGG: hsa04512
Tumor_cell	TAM-SPP1	COL4A2	CD44	0.013765	0	COL4A2_CD44	COL4A2 - CD44	COLLAGEN	ECM-Receptor	KEGG: hsa04512
Tumor_cell	TAM-SPP1	ANXA1	FPR2_LXA4	0.013005	0.03	ANXA1_FPR2_LXA4	ANXA1 - (FPR2+LXA4)	ANXA1	Cell-Cell Contact	PMID: 22610094
Tumor_cell	TAM-SPP1	LAMA5	ITGA1_ITGB1	0.012695	0	LAMA5_ITGA1_ITGB1	LAMA5 - (ITGA1+ITGB1)	LAMININ	ECM-Receptor	KEGG: hsa04512
Tumor_cell	TAM-SPP1	LAMB3	ITGAV_ITGB8	0.012608	0	LAMB3_ITGAV_ITGB8	LAMB3 - (ITGAV+ITGB8)	LAMININ	ECM-Receptor	KEGG: hsa04512
Tumor_cell	TAM-SPP1	SEMA3C	NRP1_PLXNA3	0.01223	0	SEMA3C_NRP1_PLXNA3	SEMA3C - (NRP1+PLXNA3)	SEMA3	Secreted Signaling	PMID: 27533782
Tumor_cell	TAM-SPP1	LAMC1	ITGA1_ITGB1	0.012214	0	LAMC1_ITGA1_ITGB1	LAMC1 - (ITGA1+ITGB1)	LAMININ	ECM-Receptor	KEGG: hsa04512
Tumor_cell	TAM-SPP1	COL1A1	CD44	0.012179	0	COL1A1_CD44	COL1A1 - CD44	COLLAGEN	ECM-Receptor	KEGG: hsa04512
Tumor_cell	TAM-SPP1	THBS1	ITGA3_ITGB1	0.011853	0.04	THBS1_ITGA3_ITGB1	THBS1 - (ITGA3+ITGB1)	THBS	ECM-Receptor	KEGG: hsa04512
Tumor_cell	TAM-SPP1	CCL15	CCR1	0.011573	0	CCL15_CCR1	CCL15 - CCR1	CCL	Secreted Signaling	KEGG: hsa04060
Tumor_cell	TAM-SPP1	SEMA3C	PLXND1	0.010489	0	SEMA3C_PLXND1	SEMA3C - PLXND1	SEMA3	Secreted Signaling	PMID: 27533782
Tumor_cell	TAM-SPP1	LAMB2	ITGA3_ITGB1	0.010397	0	LAMB2_ITGA3_ITGB1	LAMB2 - (ITGA3+ITGB1)	LAMININ	ECM-Receptor	KEGG: hsa04512
Tumor_cell	TAM-SPP1	LAMC2	ITGAV_ITGB8	0.010305	0	LAMC2_ITGAV_ITGB8	LAMC2 - (ITGAV+ITGB8)	LAMININ	ECM-Receptor	KEGG: hsa04512

Tumor_cell	TAM-SPP1	SEMA3C	NRP2_PLXNA3	0.009159	0	SEMA3C_NRP2_PLXNA3	SEMA3C - (NRP2+PLXNA3)	SEMA3	Secreted Signaling	PMID: 27533782
Tumor_cell	TAM-SPP1	LAMB2	ITGA1_ITGB1	0.008974	0	LAMB2_ITGA1_ITGB1	LAMB2 - (ITGA1+ITGB1)	LAMININ	ECM-Receptor	KEGG: hsa04512
Tumor_cell	TAM-SPP1	SEMA3B	NRP1_PLXNA3	0.008735	0	SEMA3B_NRP1_PLXNA3	SEMA3B - (NRP1+PLXNA3)	SEMA3	Secreted Signaling	PMID: 27533782
Tumor_cell	TAM-SPP1	FN1	ITGA5_ITGB1	0.008627	0	FN1_ITGA5_ITGB1	FN1 - (ITGA5+ITGB1)	FN1	ECM-Receptor	KEGG: hsa04512
Tumor_cell	TAM-SPP1	COL1A2	CD44	0.008216	0	COL1A2_CD44	COL1A2 - CD44	COLLAGEN	ECM-Receptor	KEGG: hsa04512
Tumor_cell	TAM-SPP1	FN1	SDC4	0.008019	0	FN1_SDC4	FN1 - SDC4	FN1	ECM-Receptor	KEGG: hsa04512
Tumor_cell	TAM-SPP1	COL9A3	SDC4	0.007479	0	COL9A3_SDC4	COL9A3 - SDC4	COLLAGEN	ECM-Receptor	KEGG: hsa04512
Tumor_cell	TAM-SPP1	LAMB1	ITGAV_ITGB8	0.007332	0	LAMB1_ITGAV_ITGB8	LAMB1 - (ITGAV+ITGB8)	LAMININ	ECM-Receptor	KEGG: hsa04512
Tumor_cell	TAM-SPP1	COL4A1	CD44	0.007068	0	COL4A1_CD44	COL4A1 - CD44	COLLAGEN	ECM-Receptor	KEGG: hsa04512
Tumor_cell	TAM-SPP1	SEMA3B	NRP2_PLXNA3	0.006536	0	SEMA3B_NRP2_PLXNA3	SEMA3B - (NRP2+PLXNA3)	SEMA3	Secreted Signaling	PMID: 27533782
Tumor_cell	TAM-SPP1	EFNB2	EPHB4	0.006111	0	EFNB2_EPHB4	EFNB2 - EPHB4	EPHB	Cell-Cell Contact	PMID: 15114347
Tumor_cell	TAM-SPP1	LAMA3	ITGAV_ITGB8	0.006023	0	LAMA3_ITGAV_ITGB8	LAMA3 - (ITGAV+ITGB8)	LAMININ	ECM-Receptor	KEGG: hsa04512
Tumor_cell	TAM-SPP1	EFNB1	EPHB4	0.005925	0	EFNB1_EPHB4	EFNB1 - EPHB4	EPHB	Cell-Cell Contact	PMID: 15114347

Tumor_cell	TAM-SPP1	HBEGF	EGFR	0.005906	0	HBEGF_EGFR	HBEGF - EGFR	EGF	Secreted Signaling	KEGG: hsa04012
Tumor_cell	TAM-SPP1	LAMA5	ITGAV_ITGB8	0.004968	0	LAMA5_ITGAV_ITGB8	LAMA5 - (ITGAV+ITGB8)	LAMININ	ECM-Receptor	KEGG: hsa04512
Tumor_cell	TAM-SPP1	AREG	EGFR	0.004856	0	AREG_EGFR	AREG - EGFR	EGF	Secreted Signaling	KEGG: hsa04012
Tumor_cell	TAM-SPP1	JAG1	NOTCH2	0.004809	0	JAG1_NOTCH2	JAG1 - NOTCH2	NOTCH	Cell-Cell Contact	PMID: 22353464
Tumor_cell	TAM-SPP1	LAMC1	ITGAV_ITGB8	0.004779	0	LAMC1_ITGAV_ITGB8	LAMC1 - (ITGAV+ITGB8)	LAMININ	ECM-Receptor	KEGG: hsa04512
Tumor_cell	TAM-SPP1	GAS6	TYRO3	0.004775	0	GAS6_TYRO3	GAS6 - TYRO3	GAS	Secreted Signaling	PMID: 27801848
Tumor_cell	TAM-SPP1	ULBP1	KLRK1_HCST	0.004555	0	ULBP1_KLRK1_HCST	ULBP1 - (KLRK1+HCST)	NKG2D	Cell-Cell Contact	PMID: 24223577
Tumor_cell	TAM-SPP1	CD6	ALCAM	0.004517	0.03	CD6_ALCAM	CD6 - ALCAM	CD6	Cell-Cell Contact	PMID: 23602662
Tumor_cell	TAM-SPP1	COL4A2	SDC4	0.004377	0	COL4A2_SDC4	COL4A2 - SDC4	COLLAGEN	ECM-Receptor	KEGG: hsa04512
Tumor_cell	TAM-SPP1	FN1	ITGAV_ITGB1	0.004256	0.01	FN1_ITGAV_ITGB1	FN1 - (ITGAV+ITGB1)	FN1	ECM-Receptor	KEGG: hsa04512
Tumor_cell	TAM-SPP1	EFNA4	EPHA1	0.003966	0	EFNA4_EPHA1	EFNA4 - EPHA1	EPHA	Cell-Cell Contact	PMID: 15114347
Tumor_cell	TAM-SPP1	COL1A1	SDC4	0.003868	0	COL1A1_SDC4	COL1A1 - SDC4	COLLAGEN	ECM-Receptor	KEGG: hsa04512
Tumor_cell	TAM-SPP1	DLL4	NOTCH2	0.003585	0	DLL4_NOTCH2	DLL4 - NOTCH2	NOTCH	Cell-Cell Contact	PMID: 22353464

Tumor_cell	TAM-SPP1	LAMB2	ITGAV_ITGB8	0.003504	0	LAMB2_ITGAV_ITGB8	LAMB2 - (ITGAV+ITGB8)	LAMININ	ECM-Receptor	KEGG: hsa04512
Tumor_cell	TAM-SPP1	AREG	EGFR_ERBB2	0.003448	0.01	AREG_EGFR_ERBB2	AREG - (EGFR+ERBB2)	EGF	Secreted Signaling	KEGG: hsa04012
Tumor_cell	TAM-SPP1	GAS6	AXL	0.003199	0.01	GAS6_AXL	GAS6 - AXL	GAS	Secreted Signaling	PMID: 27801848
Tumor_cell	TAM-SPP1	FN1	ITGA3_ITGB1	0.003126	0.01	FN1_ITGA3_ITGB1	FN1 - (ITGA3+ITGB1)	FN1	ECM-Receptor	KEGG: hsa04512
Tumor_cell	TAM-SPP1	KITLG	KIT	0.002978	0	KITL_KIT	KITL - KIT	KIT	Secreted Signaling	KEGG: hsa04080
Tumor_cell	TAM-SPP1	COL9A3	ITGA3_ITGB1	0.002915	0	COL9A3_ITGA3_ITGB1	COL9A3 - (ITGA3+ITGB1)	COLLAGEN	ECM-Receptor	KEGG: hsa04512
Tumor_cell	TAM-SPP1	PGF	FLT1	0.002859	0	PGF_VEGFR1	PGF - VEGFR1	VEGF	Secreted Signaling	KEGG: hsa04370; PMID: 16633338
Tumor_cell	TAM-SPP1	TNC	SDC4	0.002799	0	TNC_SDC4	TNC - SDC4	TENASCIN	ECM-Receptor	KEGG: hsa04512
Tumor_cell	TAM-SPP1	SEMA4G	PLXNB2	0.002666	0	SEMA4G_PLXNB2	SEMA4G - PLXNB2	SEMA4	Cell-Cell Contact	PMID: 27533782
Tumor_cell	TAM-SPP1	COL1A2	SDC4	0.002603	0	COL1A2_SDC4	COL1A2 - SDC4	COLLAGEN	ECM-Receptor	KEGG: hsa04512
Tumor_cell	TAM-SPP1	EFNA3	EPHA1	0.00257	0	EFNA3_EPHA1	EFNA3 - EPHA1	EPHA	Cell-Cell Contact	PMID: 15114347
Tumor_cell	TAM-SPP1	VEGFB	FLT1	0.002543	0	VEGFB_VEGFR1	VEGFB - VEGFR1	VEGF	Secreted Signaling	KEGG: hsa04370; PMID: 16633338
Tumor_cell	TAM-SPP1	COL9A3	ITGA1_ITGB1	0.002513	0	COL9A3_ITGA1_ITGB1	COL9A3 - (ITGA1+ITGB1)	COLLAGEN	ECM-Receptor	KEGG: hsa04512

Tumor_cell	TAM-SPP1	COL4A1	SDC4	0.002237	0	COL4A1_SDC4	COL4A1 - SDC4	COLLAGEN	ECM-Receptor	KEGG: hsa04512
Tumor_cell	TAM-SPP1	COL4A2	ITGA3_ITGB1	0.001703	0	COL4A2_ITGA3_ITGB1	COL4A2 - (ITGA3+ITGB1)	COLLAGEN	ECM-Receptor	KEGG: hsa04512
Tumor_cell	TAM-SPP1	PROS1	TYRO3	0.001689	0	PROS1_TYRO3	PROS1 - TYRO3	PROS	Secreted Signaling	PMID: 30501104
Tumor_cell	TAM-SPP1	COL1A1	ITGA3_ITGB1	0.001504	0	COL1A1_ITGA3_ITGB1	COL1A1 - (ITGA3+ITGB1)	COLLAGEN	ECM-Receptor	KEGG: hsa04512
Tumor_cell	TAM-SPP1	ULBP1	KLRK1	0.00149	0	ULBP1_KLRK1	ULBP1 - KLRK1	MHC-I	Cell-Cell Contact	PMID: 24223577
Tumor_cell	TAM-SPP1	COL4A2	ITGA1_ITGB1	0.001468	0	COL4A2_ITGA1_ITGB1	COL4A2 - (ITGA1+ITGB1)	COLLAGEN	ECM-Receptor	KEGG: hsa04512
Tumor_cell	TAM-SPP1	COL1A1	ITGA1_ITGB1	0.001297	0	COL1A1_ITGA1_ITGB1	COL1A1 - (ITGA1+ITGB1)	COLLAGEN	ECM-Receptor	KEGG: hsa04512
Tumor_cell	TAM-SPP1	SEMA5A	PLXNA3	0.001233	0	SEMA5A_PLXNA3	SEMA5A - PLXNA3	SEMA5	Cell-Cell Contact	PMID: 27533782
Tumor_cell	TAM-SPP1	PROS1	AXL	0.00113	0	PROS1_AXL	PROS1 - AXL	PROS	Secreted Signaling	PMID: 29531161
Tumor_cell	TAM-SPP1	FN1	ITGAV_ITGB8	0.001048	0.04	FN1_ITGAV_ITGB8	FN1 - (ITGAV+ITGB8)	FN1	ECM-Receptor	KEGG: hsa04512
Tumor_cell	TAM-SPP1	COL1A2	ITGA3_ITGB1	0.001011	0.01	COL1A2_ITGA3_ITGB1	COL1A2 - (ITGA3+ITGB1)	COLLAGEN	ECM-Receptor	KEGG: hsa04512
Tumor_cell	TAM-SPP1	COL9A3	ITGAV_ITGB8	0.000977	0	COL9A3_ITGAV_ITGB8	COL9A3 - (ITGAV+ITGB8)	COLLAGEN	ECM-Receptor	KEGG: hsa04512
Tumor_cell	TAM-SPP1	COL1A2	ITGA1_ITGB1	0.000872	0.01	COL1A2_ITGA1_ITGB1	COL1A2 - (ITGA1+ITGB1)	COLLAGEN	ECM-Receptor	KEGG: hsa04512

Tumor_cell	TAM-SPP1	COL4A1	ITGA3_ITGB1	0.000869	0	COL4A1_ITGA3_ITGB1	COL4A1 - (ITGA3+ITGB1)	COLLAGEN	ECM-Receptor	KEGG: hsa04512
Tumor_cell	TAM-SPP1	COL9A3	ITGA11_ITGB1	0.000793	0	COL9A3_ITGA11_ITGB1	COL9A3 - (ITGA11+ITGB1)	COLLAGEN	ECM-Receptor	KEGG: hsa04512
Tumor_cell	TAM-SPP1	COL4A1	ITGA1_ITGB1	0.000749	0	COL4A1_ITGA1_ITGB1	COL4A1 - (ITGA1+ITGB1)	COLLAGEN	ECM-Receptor	KEGG: hsa04512
Tumor_cell	TAM-SPP1	COL4A2	ITGAV_ITGB8	0.00057	0	COL4A2_ITGAV_ITGB8	COL4A2 - (ITGAV+ITGB8)	COLLAGEN	ECM-Receptor	KEGG: hsa04512
Tumor_cell	TAM-SPP1	ENTPD1	ADORA2A	0.000529	0	ENTPD1_ADORA2A	ENTPD1 - ADORA2A	CD39	Cell-Cell Contact	PMID: 21677139
Tumor_cell	TAM-SPP1	COL1A1	ITGAV_ITGB8	0.000504	0	COL1A1_ITGAV_ITGB8	COL1A1 - (ITGAV+ITGB8)	COLLAGEN	ECM-Receptor	KEGG: hsa04512
Tumor_cell	TAM-SPP1	CADM1	CADM1	0.000463	0.03	CADM1_CADM1	CADM1 - CADM1	CADM	Cell-Cell Contact	PMID: 24503895
Tumor_cell	TAM-SPP1	COL4A2	ITGA11_ITGB1	0.000463	0	COL4A2_ITGA11_ITGB1	COL4A2 - (ITGA11+ITGB1)	COLLAGEN	ECM-Receptor	KEGG: hsa04512
Tumor_cell	TAM-SPP1	COL1A1	ITGA11_ITGB1	0.000409	0	COL1A1_ITGA11_ITGB1	COL1A1 - (ITGA11+ITGB1)	COLLAGEN	ECM-Receptor	KEGG: hsa04512
Tumor_cell	TAM-SPP1	COL1A2	ITGAV_ITGB8	0.000339	0.01	COL1A2_ITGAV_ITGB8	COL1A2 - (ITGAV+ITGB8)	COLLAGEN	ECM-Receptor	KEGG: hsa04512
Tumor_cell	TAM-SPP1	NECTIN1	CD96	0.000332	0	NECTIN1_CD96	NECTIN1 - CD96	NECTIN	Cell-Cell Contact	PMID: 23027581
Tumor_cell	TAM-SPP1	COL4A1	ITGAV_ITGB8	0.000291	0	COL4A1_ITGAV_ITGB8	COL4A1 - (ITGAV+ITGB8)	COLLAGEN	ECM-Receptor	KEGG: hsa04512
Tumor_cell	TAM-SPP1	COL1A2	ITGA11_ITGB1	0.000275	0.04	COL1A2_ITGA11_ITGB1	COL1A2 - (ITGA11+ITGB1)	COLLAGEN	ECM-Receptor	KEGG: hsa04512

Tumor_cell	TAM-SPP1	COL4A1	ITGA11_ITGB1	0.000236	0	COL4A1_ITGA11_ITGB1	COL4A1 - (ITGA11+ITGB1)	COLLAGEN	ECM-Receptor	KEGG: hsa04512

Supplementary Table S2. Information of the clinical tissue microarray sample (n=42).

**Characteristic**	**CRC**, N = 38[1]	**Control**, N = 4[1]
Age	54 (50, 68)	NA
Unknown	5	4
Gender		
female	17 (46%)	0 (0%)
male	20 (54%)	4 (100%)
Unknown	1	0
Tissue source		
colon	32 (84%)	4 (100%)
Metastasis-liver	3 (7.9%)	0 (0%)
Metastasis-lung	1 (2.6%)	0 (0%)
Metastasis-ovarian	2 (5.3%)	0 (0%)
Tissue Type		
normal tissue	0 (0%)	4 (100%)
adenocarcinoma	26 (68%)	0 (0%)
tubular adenomas	3 (7.9%)	0 (0%)
tubulovillous adenoma with intraepithelial neoplasia	3 (7.9%)	0 (0%)
metastatic adenocarcinoma	6 (16%)	0 (0%)
Pathological_staging		
II grade	11 (61%)	
II-III grade	2 (11%)	
low grade	5 (28%)	
Unknown	20	
Metastasis		
No	27 (71%)	

**Characteristic**	**CRC**, N = 38[1]	**Control**, N = 4[1]
Liver	8 (21%)	
Lung	1 (2.6%)	
ovarian	2 (5.3%)	
Unknown	0	
T		
not applicable	0 (0%)	
T2	2 (20%)	
T3	8 (80%)	
Unknown	28	
N		
N0	9 (35%)	
N1a	7 (27%)	
N1b	3 (12%)	
N2a	3 (12%)	
N2b	4 (15%)	
not applicable	0 (0%)	
Unknown	12	
M		
M0	21 (66%)	
M1	6 (19%)	
M1a	5 (16%)	
not applicable	0 (0%)	
Unknown	6	
AJCC-7th-staging		
1	2 (6.3%)	
2	5 (16%)	

**Characteristic**	**CRC**, N = 38[1]	**Control**, N = 4[1]
2A	2 (6.3%)	
3	8 (25%)	
3B	4 (13%)	
4	6 (19%)	
4A	5 (16%)	
Unknown	6	
Tumor_volumns	50 (25, 116)cm^3	
Unknown	6	

[1]Median (Interquartile range); n (%)
